# LITO: Lemur-Inspired Task Offloading for Edge–Fog–Cloud Continuum Systems

**DOI:** 10.3390/s26051497

**Published:** 2026-02-27

**Authors:** Asma Almulifi, Heba Kurdi

**Affiliations:** Computer Science Department, College of Computer and Information Sciences, King Saud University, Riyadh 11451, Saudi Arabia

**Keywords:** edge–fog–cloud continuum, fog computing, edge computing, task offloading, resource management, Internet of Things, supervised policy learning

## Abstract

Edge, fog, and cloud continuum architectures that interconnect resource-constrained devices, intermediate edge servers, and remote cloud data centers face persistent challenges in handling heterogeneous and latency-sensitive workloads while reducing energy consumption and improving resource utilization. Classical task offloading approaches either rely on static heuristics, which lack adaptability to dynamic conditions, or on metaheuristic optimizers, which often incur high computational overhead and centralized coordination. This paper proposes LITO, a lemur-inspired task offloading algorithm for edge, fog, and cloud continuum systems that models the infrastructure as a social system in which computing nodes assume distinct roles that mirror lemur social hierarchies. Building on an abstracted model of lemur group behavior, LITO incorporates two key lemur-inspired mechanisms: an energy-aware task assignment mechanism based on sun basking, a thermoregulation behavior in which lemurs seek favorable warm spots, mapped here to selecting energetically efficient execution nodes, and a cooperative scheduling policy based on huddling, group clustering under stress, mapped here to sharing load among overloaded nodes. These mechanisms are combined with a continual supervised policy-learning layer with contextual bandit feedback that refines offloading decisions from online feedback. The resulting multi-objective formulation jointly minimizes energy consumption and deadline violations while maximizing resource utilization and throughput under high-load conditions in the edge and fog segment of the continuum. Simulations under diverse workload regimes and task complexities show that LITO outperforms representative multi-objective offloading baselines in terms of energy consumption, resource utilization, latency, Service Level Agreement (SLA) violations, and throughput in congested scenarios.

## 1. Introduction

Fog computing has emerged as a pivotal paradigm that extends the cloud model by decentralizing storage, computation, and networking resources toward the periphery of Internet of Things (IoT) environments. Within such ecosystems, smart sensors deployed across domains such as smart healthcare, smart transportation, and smart cities continuously generate massive volumes of heterogeneous data. Even though cloud computing offers scalable resources, its dependence on centralized infrastructures inevitably incurs bandwidth congestion, high latency, and heightened data privacy concerns [1,2]. To mitigate these challenges, computation has progressively migrated closer to the edge. While edge devices (EDs) provide preliminary processing and rapid responsiveness, their limited resources impose fundamental restrictions on sustained performance and scalability. Fog computing therefore emerges as an intermediate layer that bridges edge and cloud environments.

Fog computing extends cloud computation, storage, and networking capabilities to the network edge, thereby addressing latency and Quality of Service (QoS) challenges [3,4]. It strategically deploys geo-distributed fog nodes such as routers, switches, and access points between IoT devices and centralized cloud infrastructure, facilitating local data storage, computation, and resource orchestration while simultaneously alleviating congestion. Architecturally, fog/edge–cloud collaborative computing is commonly described as a multi-layer model spanning end devices, nearby fog/edge nodes, and the back-end cloud [5]. This layered design enables efficient local processing while maintaining seamless cloud connectivity, offering advantages such as low latency, interoperability, scalability, real-time interaction, and support for heterogeneous devices [6]. By relocating computation and decision-making closer to the data source, fog computing enables faster responses, optimized bandwidth utilization, and improved QoS. Reflecting this importance, the global fog computing market, valued at USD 448 million in 2024, is expected to reach USD 5.41 billion by 2035, propelled by industrial automation and IoT adoption [7]. Furthermore, fog computing supports application domains unsuitable for cloud-only solutions, particularly those requiring geographically distributed systems, ultra-low latency, and large-scale adaptive control [8].

To enable such diverse applications, the fog network encompasses heterogeneous devices endowed with computation, storage, and communication capabilities. However, the inherently dynamic nature of fog environments—compounded by device mobility and fluctuating workloads—makes resource management a significant challenge. Effective resource management is essential to fully leverage the processing power of fog and improve overall performance [9]. Within this framework, task offloading plays a vital role, allowing IoT end devices to delegate workloads to nearby fog nodes for remote computation, thereby reducing latency while conserving energy. As the proliferation of IoT devices increases, maintaining stringent QoS parameters such as response time, throughput, and resource utilization becomes critical [4]. Offloading strategies seek to optimize computing performance by judiciously selecting appropriate layers, fog or edge, depending on energy efficiency, processing demand, and cost-effectiveness [10]. However, the offloading process remains profoundly complex, requiring effective fog node selection to balance resources and ensure seamless operations [11]. Consequently, contemporary research in fog computing focuses on optimizing objectives such as energy minimization, service delay reduction, and bandwidth efficiency, while ensuring compliance with constraints imposed by QoS guarantees, power limitations, and application-specific requirements [12].

While conventional heuristic-based offloading strategies often fall short in dynamic and heterogeneous environments [13], metaheuristic algorithms have shown greater promise due to their ability to explore large solution spaces and balance exploration and exploitation [14]. Metaheuristics, many of which are inspired by biological or physical processes, offer adaptive and scalable solutions to optimization problems, supporting improved responsiveness and better resource utilization [15,16,17]. However, scaling these methods in large fog networks introduces new limitations. Traditional algorithms such as Genetic Algorithms (GA), Particle Swarm Optimization (PSO), Grey Wolf Optimization (GWO), and Simulated Annealing (SA) may suffer from low convergence speed, susceptibility to local optima, and computational inefficiency in real-time settings [18]. Moreover, many approaches assume relatively stable conditions or require extensive tuning/training to remain effective as network states and resources vary, which can hinder adaptability and scalability in real-world deployments [19,20].

Motivated by these gaps, we introduce the Lemur-Inspired Task Offloading (LITO) framework, which is a decentralized and adaptive approach. The term decentralized in LITO indicates the distribution of decision-making authority across Primary Nodes (PNs), Support Nodes (SNs), and EDs, in which each tier takes autonomous yet coordinated decisions without depending on a single centralized optimizer. EDs independently find local execution or offloading through Very fast decision trees (VFDT) classifiers, which are trained on streaming task features. By utilizing aggregated telemetry and the continual supervised policy-learning formulation with contextual bandit feedback (CSPL-CB) learner, PNs make segment-level global routing decisions. In contrast, SNs implement hybrid Earliest Deadline First–Dynamic Resource Scheduling (EDF–DRS) scheduling locally. This layered autonomy minimizes coordination overhead and avoids the dependence on global recomputation. Adaptiveness in LITO is attained through feedback-driven policy refinement and multi-level continual learning. VFDT updates local offloading decisions incrementally under varying workload conditions, whereas CSPL-CB at PNs refines offloading policies continuously using oracle supervision and contextual bandit feedback under non-stationary conditions. Depending on real-time resource availability and urgency factors, SNs adjust scheduling priorities dynamically. The feedback and adaptation module facilitates policy evolution under fluctuating network bandwidth, energy levels, and node utilization. These mechanisms enable LITO to preserve deadline adherence, energy efficiency, and workload balance across evolving fog infrastructures.

We first formulated the task offloading problem as a constrained multi-objective optimization problem that jointly accounts for energy consumption, end-to-end latency, and resource–utilization balance under deadline and capacity constraints. We then evaluated LITO against representative multi-objective baselines within a fog-computing simulation environment by designing scenarios that vary workload intensity, task complexity, and system configuration. Finally, we complemented these external baselines with an ablation study that isolates the effect of LITO design choices (local-only execution, full offloading, hybrid edge policy, support-node-only execution, and the proposed edge support collaboration), providing a focused yet comprehensive evaluation of the proposed framework. Across these experiments, the proposed LITO approach demonstrates up to 60% reduction in energy consumption and over 30% improvement in resource utilization compared to state-of-the-art multi-objective offloading baselines, alongside lower latency and higher SLA compliance under stress-tested fog scenarios.

The main contributions of this work are outlined as follows:

- Lemur-inspired task offloading framework: We proposed a Lemur-Inspired Task Offloading (LITO) framework that models fog environments as social systems. By assigning role-based hierarchies to Edge Devices (EDs), Support Nodes (SNs), and Primary Nodes (PNs), the system enables decentralized, adaptive task distribution inspired by lemur group behaviors.
- Hybrid local learning and scheduling: We developed an integrated hybrid learning architecture that combines Very Fast Decision Trees (VFDT) at the ED level for incremental, local decision-making with a hybrid Earliest Deadline First–Dynamic Resource Scheduling (EDF–DRS) policy at the SN level, improving responsiveness under varying workload conditions.
- Continual policy learning with contextual bandits: We employed a continual supervised policy-learning formulation with contextual bandit feedback (CSPL-CB) at the PN level to optimize global offloading policies over time. This approach supports learning continuity and adaptability to evolving network dynamics, enhancing long-term policy efficiency.
- Comprehensive empirical evaluation: We conducted a comprehensive empirical evaluation across varying workloads, task complexities, and system configurations.

This paper is organized as follows. Section 2 reviews the related literature. Section 3 details the proposed system design. Section 4 outlines the evaluation methodology, and Section 5 presents the experimental results. Finally, Section 6 concludes the paper and discusses directions for future research.

## 2. Literature Review

Task offloading has attracted significant research attention because of the increasing demand for latency-sensitive and resource-constrained applications. Several studies address these challenges using metaheuristic-based optimization techniques that search for near-optimal offloading strategies under multiple performance objectives.

### 2.1. Metaheuristics-Based Task Offloading Approaches

Existing studies on task offloading in edge and fog environments can be classified into (i) trajectory-aware energy optimization, (ii) multi-objective metaheuristic optimization, and (iii) workflow and digital twin-assisted optimization frameworks.

Unmanned Aerial Vehicle (UAV)-assisted MEC systems present unique limitations because of obstacle avoidance, limited battery storage, and dynamic maneuverability demands. Hence, a joint optimization framework [21] is proposed to improve energy efficiency by simultaneously enhancing user task offloading rates and UAV trajectories. This framework combines hybrid alternating metaheuristics and transformation techniques to effectively enable obstacle-free UAV navigation and reduce total energy consumption. However, the offloading tasks are assumed with fixed bandwidth and computational requirements, overlooking real-world heterogeneity in which tasks may differ in deadlines, priority, and dependencies across devices.

User equipment-driven offloading decisions become a critical challenge because of high energy consumption and congestion at the edge. To address these, offloading is considered as a constrained multi-objective optimization problem in [22], and metaheuristic techniques, like Tug of War Optimization, Hybrid Improved Whale Optimization, and Adaptive Equilibrium Optimization, are employed that minimize execution delays and energy utilization with improved task satisfaction. However, it does not explicitly consider multi-user task offloading circumstances. To address this problem, a dynamic multi-user, multi-server MEC system is developed in [23] along with stochastic task arrivals. A PSO-based algorithm aids in minimizing average task delay via efficient load balancing and adaptive server selection. Even though makespan and energy are optimized, the trade-off between them is not investigated under diverse application requirements.

In order to optimize multiple objectives like energy consumption, makespan, and resource utilization, a quantum-inspired PSO approach [24] is proposed for workflow scheduling in edge environments. However, the PSO parameters, such as learning factors, swarm size, and iterations, are fixed across diverse experiments. To tackle the task of offloading challenges on the Internet of Drones (IoD), PSO is integrated with fog base stations in [25] to minimize transmission delays and enhance processing and storage capacity. However, it leverages static acceleration coefficients and a fixed number of iterations that may not efficiently adapt to real-time fluctuations.

In IIoT, a multi-objective algorithm task offloading, named ODTRA [26], is proposed that utilizes probabilistic recursive local search (PRLS) and the water cycle metaphor (WCM) to minimize the execution time. Digital twin is leveraged for real-time monitoring and predictive modelling, while allowing intelligent and proactive resource allocation and task offloading. It improves performance by minimizing latency, enhancing energy efficiency, and ensuring QoS. However, fixed parameters such as noise, bandwidth, distance, and energy weights do not consider real-world variations.

### 2.2. Metaheuristics-Based Task Offloading Approaches in Fog Computing

Existing studies on metaheuristic-based task offloading in fog environments can be divided into six groups on the basis of their optimization objectives and decision strategies. They are (i) latency–load balancing optimization, (ii) multi-objective system-wide optimization, (iii) joint offloading–scheduling integration, (iv) context-aware optimization approaches, (v) hybrid and multi-strategy metaheuristic frameworks, and (vi) distributed and online optimization approaches.

Several studies [27,28,29] tackle the problem of balancing task offloading time, latency, and workload distribution in three-tier IoT–fog–cloud architectures. By identifying the inefficiency of cloud-only offloading in latency-sensitive IoT applications, a hybrid framework is proposed in [27] using the Flamingo Search Algorithm (FSA) for initial exploration and the Honey Badger Algorithm (HBA) for refinement. This framework integrates the benefits of both algorithms to construct an optimized offloading framework with reduced latency and improved workload balance but fails to scale efficiently. Similarly, in [28], offloading is formulated as a multi-objective optimization problem, concentrating on resource utilization and delay reduction in fog computing. The Discrete Jaya Algorithm (DJA) is utilized with a task migration scheme, while ensuring resilience and improving adaptability and efficiency. However, the NP-hard nature makes convergence slow. Processing and energy constraints in end devices necessitate effective task offloading across IoT–fog–cloud. Existing approaches focus on localized optimization rather than system-wide optimization. A task offloading model [30] has been developed using an improved non-dominated sorting crow search algorithm (INSCSA) with multiple objectives such as response time, energy, cost, and availability. It attains system-wide optimization with improved overall efficiency. Both studies enable efficient exploration and exploitation, thus enhancing resource utilization and system responsiveness within dynamic fog environments.

Studies [31,32,33] underscore the interdependence between scheduling and offloading tasks within heterogeneous fog–cloud systems. In [31], the trade-off between delay and energy is highlighted in cloud-fog systems in which task execution order directly influences stability. A Multi-objective Arithmetic Optimization Algorithm (MoAOA) is developed with a personalized fitness function by considering energy, delay, and task priority. It achieves balanced offloading and efficient scheduling, dynamically adapting to varied workloads. However, it is computationally expensive when the network size increases, resulting in extended convergence time for large-scale deployments. To address the issues related to high latency, inefficient resource allocation, and energy consumption in fog computing, offloading is applied hierarchically in [32], in which tasks are transferred from devices to fog nodes, higher-capacity fog nodes, and then to the cloud when needed. A hybrid GA-SA-GWO is leveraged to optimize latency, task scheduling, energy utilization, and overall system performance. Even though hybrid metaheuristics are leveraged, the main contribution is in combining hierarchical offloading decisions and scheduling. However, this hybrid algorithm will increase computational complexity and delay the offloading decision-making process in highly dynamic environments. To tackle these challenges, GA Hybrid-Fog is proposed in [33], which combines GA with fog base stations and fog UAVs to optimize both transmission and fog computing latency. It considers both static and mobile fog resources but does not address heterogeneous task requirements or task priority management. This requirement influences system reliability when high-priority tasks are delayed due to low-priority jobs. The model assumes ideal conditions for noise power, transmission bandwidth, and processing cycles.

Context-aware optimization methods [34,35,36] adapt task offloading strategies for specific workload characteristics or application scenarios. In [34], smart ant colony optimization (SACO) is developed to address the latency issues in IoT-sensor-based applications. Notable reductions in offloading delay are achieved by comparing SACO with throttled scheduling, Round Robin, and other bio-inspired algorithms. A study [35] has extended this task offloading process to workflow applications within heterogeneous fog–cloud infrastructures. Fuzzy dominance-based clustering is leveraged along with the hybrid Harmony Search Algorithm (HSA) and GA for task scheduling. It effectively balances makespan, cost, and resource utilization; however, it assumes static task arrival patterns in workflows. For critical task execution, a two-stage framework [36] is proposed with enhanced task offloading (ETO). It chooses the computing layer, which is followed by the multi-objective firefly algorithm (MFA) for device selection in the selected layer. This layered approach aids in minimizing energy consumption and delay while improving reliability. However, the study does not consider the resource heterogeneity across fog, edge, and cloud layers, affecting real-world performance.

Existing studies [37,38,39] have explored hybrid and multi-strategy optimizers to resolve the shortcomings of conventional metaheuristic algorithms. A logarithm Mayfly Algorithm (lMA) [37] is introduced for bag-of-tasks applications within fog systems. 1MA effectively balances local exploitation and global exploration by refining global coefficients and individual learning. It proves efficiency gains in cost and execution time, but it fails to integrate strict task deadlines, budget limits, varying network latencies, or fluctuating energy availability into its optimization process. In [38], an Integrated Snake Optimizer (ISO) is utilized along with circle map initialization, elite reverse learning, and synergistic global search to improve solution diversity and convergence speed and reduce costs and delays. Even though it addresses independent bag-of-tasks applications, it may not generalize well to interdependent or workflow-based tasks common in real IoT–fog–cloud applications. A multi-objective optimization problem is solved using Non-dominated Sorting GA II (NSGA-II) and the Bees Algorithm [39] within mobile fog systems. A balanced trade-off between energy consumption and task execution delay is ensured, while achieving robustness in dynamic mobile environments. However, it depends on hierarchical agglomerative clustering, which incurs high time complexity. Moreover, it is computationally expensive on large networks, even if clustering is assumed to be infrequently executed.

Studies [40,41] have explored distributed and online optimization models. A fully distributed computation offloading algorithm [40] is proposed to reach the Nash equilibrium without centralized coordination. Even though fully distributed, it assumes normal user behavior and improves execution cost, without solving hierarchical fog coordination or adaptation in case of non-stationary workloads. Similarly, a two-time-scale accuracy-aware online optimization framework [41] is proposed that depends on Lyapunov decomposition. Although it dynamically handles delay and energy constraints, its performance is affected by the dependence on centralized control and mathematically intensive optimization. To solve dynamic task offloading in fog environments, a distributed twin-delayed deep deterministic policy gradient (TD3) framework [42] is leveraged with multiple actors, enhancing learning and scalability. However, training instability occurs in the case of highly non-stationary workloads. Distributed deep reinforcement learning (µ-DDRL) [43] addresses directed acyclic graph-based service offloading through asynchronous actor–critic learning with proximal policy optimization (PPO) clipping and V-trace. Although it enables faster convergence and better adaptability, the decision-time overhead is higher. Deep deterministic policy gradient (DDPG)-based offloading [44] is proposed to improve delay in heterogeneous edge networks. However, its single-agent design restricts scalability and robustness in large-scale distributed settings. In contrast, LITO combines hierarchical decentralization with lightweight continual learning to support adaptive, scalable, and resource-aware task delegation across fog layers.

Existing metaheuristic-based methods depend on global optimization paradigms that lack integrated online policy refinement and isolate offloading from hierarchical coordination. Although they enhance energy and latency trade-offs, their architectural designs are inadequate for scalable, multi-tier fog systems functioning under heterogeneous and emerging workload conditions. Consequently, coordinated decision-making across edge and fog nodes remains underexplored. These structural shortcomings drive the need for a decentralized, learning-driven framework that combines hierarchical coordination with adaptive policy evolution, as realized in the proposed LITO architecture. Table 1 presents a comparative analysis of well-known metaheuristic-based task offloading approaches, highlighting their primary objectives, decision-making metrics, optimization techniques, application domains, and key limitations.

From the surveyed literature, metaheuristic-based task offloading across fog and IoT environments has achieved notable improvements in latency, energy efficiency, and workload balancing. However, several research gaps persist. First, task and environmental heterogeneity are only partially addressed. In many studies [21,25,26,32,33,36], tasks are modeled with fixed parameters and simplified timing assumptions. Strict deadline guarantees, task priorities, and inter-task dependencies are typically not modeled explicitly, which limits the adaptability of these approaches to diverse real-world workloads. Hybrid and multi-objective approaches [27,28,30,31,32,39,45] enhance optimization quality but often incur high computational costs or rely on complex clustering and global coordination, which constrains their applicability in large-scale deployments. Likewise, static parameterization of metaheuristic algorithms in works such as [21,24,27,29,30,31,33,34,35,39,45] (for example, fixed PSO and GA coefficients) hinders adaptability to dynamic workloads. Offloading approaches in [28,30,37,45] frequently assume stable resource conditions within each time slot or provide limited treatment of node and link failures, which can lead to inefficiencies and reduced robustness in unstable environments, especially across multiple tiers of the edge–fog–cloud continuum.

Existing studies have often optimized task offloading in isolation and overlook the interdependence between task scheduling and offloading. Studies [31,45] have considered joint optimization, but scalability and dynamic adaptation challenges remain unsolved. Advanced optimizers such as lMA [37], ISO [38], and NSGA-II with Bees [39] improve convergence and robustness, yet they are computationally expensive and still do not integrate dynamic constraints such as heterogeneous deadlines, fluctuating energy availability, and varying network conditions. Most existing metaheuristic-based task offloading approaches depend on system-wide information exchange and centralized optimization with global coordination [21,24,25,26,27,28,29,30,31,32,33,34,36,37,39,45], whereas only limited works have considered decentralized [29] or partially decentralized architectures [35]. Therefore, there is still a need for a decentralized and adaptive framework that can handle heterogeneous, large-scale, and real-time environments and explicitly coordinate decisions across multiple tiers of the edge–fog–cloud continuum.

## 3. System Design

### 3.1. Problem Formulation

The LITO algorithm is designed to improve efficiency and adaptability in fog computing by dynamically offloading tasks from IoT devices to edge and fog nodes. The set of tasks is defined as *T* = {*t*_1_, *t*_2_, …, *t_n_*}, in which each task t_i_ has the following attributes: workload $W_{t_{i}}$ (CPU cycles), deadline $D_{t_{i}}$, Priority $P_{t_{i}}$, dependency set $d_{t_{i}}$ ($d_{t_{i}}$ ⊆ *T*), and energy requirement $E_{t_{i}}$. The feature vector of task t_i_ is given by,

(1) $$X_{i}=\left(W_{t_{i}},D_{t_{i}},P_{t_{i}},{E(d}_{t_{i}}),E_{t_{i}}\right)$$

As a set of task dependencies $d_{t_{i}}$ is high-dimensional categorical field, it is encoded using adjacency binary vector as ${E(d}_{t_{i}})$.

Assume the set of nodes is *M* = {*m*_1_, *m*_2_, …, *m_k_*} in which each node m_j_ has capacity C_j_ (cycles/s), energy level E_j_ (residual energy of nodes), and communication delay *L_ij_*. Let *Aj* ⊆ *T* denote the set of tasks assigned to node mj within a scheduling slot of length Ts, and let *Uj* denote the utilization of node *mj*. The main objective is to minimize the Resource Utilization Gap (RUG), which measures the difference between available computational resources and the amount actually utilized during task execution, while ensuring that deadlines, workload balance, and energy constraints are met. The objective function is given by:

(2) $$min\;J=\sum_{t_{i}\in T}\left(\alpha L_{t_{i}}+\beta E_{t_{i}}+\gamma I_{t_{i}}\right)$$

where $L_{t_{i}}$ is the end-to-end delay (including both communication and execution); $E_{t_{i}}$ is the energy consumed at the node that runs the task; $I_{t_{i}}$ quantifies the contribution of task *t_i_* to the imbalance in resource utilization across (SNs), which is directly related to the *RUG* metric. Coefficients α, β, and γ are weighting factors that reflect system priorities.

The optimization problem is formulated under practical system constraints. It ensures energy awareness, deadline compliance, feasibility, and balanced utilization across fog nodes.

- Deadline Constraint (C1): Each task needs to finish before its deadline under EDF scheduling: C1 enforces that each task must respect its deadline while being scheduled under an EDF policy:
  (3) $$C1:\;Completion\_Time(t_{i})\;\leq \;D_{t_{i}},\;\forall t_{i}\in T$$
  ensuring strict adherence to timing requirements.
- Energy-Aware Offloading Constraint (C2): A task is offloaded from an ED only when local energy $E_{t_{i}}^{local}$ is more than a predefined threshold ETh:
  (4) $$C2:\;E_{t_{i}}^{local}>E_{Th}\;(\mathrm{during}\;\mathrm{offloading})$$
  enforcing energy conservation at constrained EDs.
- Capacity Constraint (C3): The total workload allocated to an SN should not exceed its available capacity:
  (5) $$C3:\;\sum_{t_{i}\in A_{j}}W_{t_{i}}\leq C_{j}T_{s};\;\forall m_{j}\in M$$
  preventing overload within a scheduling slot Ts.
- Load Balancing Constraint (C4): It constrains the difference between the most and least utilized SNs to be within a threshold δ, thus promoting balanced resource usage:
  (6) $$C4:\;\underset{m_{j}\in M}{\max}U_{j}-\underset{m_{j}\in M}{\min}U_{j}\leq \delta$$
  promoting balanced resource allocation.
- Feasibility Constraint for Offloaded Tasks (C5): For each offloaded task, execution and communication delay must satisfy:
  (7) $$C5:\;L_{ij}+\frac{W_{t_{i}}}{C_{j}}\leq D_{t_{i}},\;\forall t_{i}\;offloaded\;to\;m_{j}$$
  guaranteeing deadline feasibility at the chosen fog node.

### 3.2. Lemur Behavior as Inspiration for PLBA

**Figure 1:**
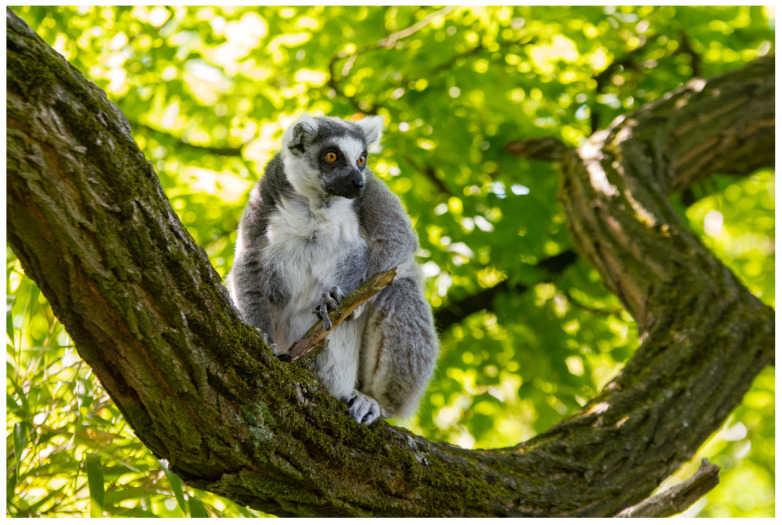
A Lemur (CC0 1.0), source: Wikimedia Commons [41].

Lemurs, shown in Figure 1, are primates native to Madagascar that live in social groups and typically inhabit forested, tree-rich environments [40]. Their societies are matriarchal, with dominant females guiding group decisions and managing access to resources. Males usually play supportive roles, such as territorial defense and conflict mediation, while offspring depend on the group for protection and learn by observing adult behavior [42].

In this work, we idealize these social dynamics into a role-based model that we call the Pure Lemur Behavior Abstraction (PLBA). PLBA is a conceptual description of how roles are distributed in a lemur group, how information flows between them, and how the group reacts to changes in the environment. PLBA acts as a conceptual behavioral framework that motivates the algorithmic mechanisms in LITO, but it does not define any learning rules or computational steps.

The LITO algorithm then instantiates PLBA in a fog-computing context. Specifically, fog components are assigned roles that mirror those in PLBA: PNs emulate the leadership role of dominant females by taking global offloading decisions; SNs correspond to supportive males that stabilize the system and help handle overflow tasks; and EDs play the role of offspring, with limited resources but the ability to make local decisions and adapt based on feedback. PLBA provides the behavioral template, while LITO defines the concrete decision rules and learning mechanisms that these nodes follow.

Individual lemur behaviors also motivate specific mechanisms in LITO. Sun basking, where lemurs conserve energy by seeking warmth, inspires an energy-aware offloading policy that prefers nodes with better energy conditions. Huddling, a group behavior used for warmth and protection during threats or cold conditions [42], motivates cooperative task redistribution and load balancing when the system is under stress or when some nodes fail. The correspondence between these lemur behaviors in PLBA and their algorithmic counterparts in LITO is summarized in Table 2.

The behavioral abstractions are mapped to explicit quantitative decision rules to reinforce the correspondence between algorithmic realization and biological inspiration.

Sun Basking to Energy-Based Offloading Rule: When an individual’s thermal energy is less than a threshold within biological systems, sun basking is triggered. In LITO, this behavior is modeled via an energy-awareness condition at EDs. Specifically, when an ED’s residual energy $E_{j}$ is less than a predefined fraction of its initial energy $\eta E_{j}^{init}$, the task is considered offload-eligible and assessed for fog-layer assignment:

(8) $$E_{j}<\eta E_{j}^{init}$$

where 0<η<1 indicates the energy preference coefficient. Amongst candidate fog nodes, selection is based on an energy-efficiency score:

(9) $$Score_{j}=\frac{C_{j}}{P_{j}^{busy}}$$

where $C_{j}$ indicates computational capacity; $P_{j}^{busy}$ indicates busy-state power consumption. Nodes that have higher scores are prioritized for task assignment.

Huddling to Utilization-Imbalance Trigger: The huddling-inspired cooperative load redistribution is performed through the utilization-imbalance constraint defined in C4 (Equation (6)), in which redistribution is activated when the utilization gap across SNs is more than threshold δ.

These threshold-based mappings create a direct and quantifiable correspondence between algorithmic decision triggers and lemur behavioral stimuli in LITO.

### 3.3. System Model

The proposed framework adopts a multi-layered infrastructure embedded in an edge–fog–cloud continuum. The system is organized into four operational layers and two auxiliary modules for coordination and performance feedback:

IoT Layer. This foundational tier is responsible for sensing, data generation, and initial communication. It includes physical devices such as sensors and actuators that produce tasks with heterogeneous workloads and latency requirements. Tasks are encapsulated into digital descriptors and sent to the edge layer for processing.

Edge Layer (Local Offloading Decision). Edge devices (EDs), representing the “ordinary lemurs” in the social model, are the first programmable tier above IoT devices. They perform lightweight preprocessing, local queuing, and initial offloading decisions based on local context (for example, queue length, residual energy, and link quality). When local execution is inefficient or infeasible, the task is packaged and forwarded to the next layer.

Fog Layer (Hierarchical Global Offloading). This layer is logically split into two sublayers that collectively implement hierarchical global offloading and scheduling:

- Upper Fog Sublayer (Primary Nodes). Primary Nodes (PNs) are high-capacity fog servers or industrial-grade controllers that maintain a global, though approximate, view of the edge–fog segment. They receive summarized state information from Support Nodes (SNs), including load conditions, energy levels, and task outcomes, and run the continual supervised policy-learning with contextual bandit feedback (CSPL-CB) mechanism to select suitable execution targets for incoming tasks.
- Lower Fog Sublayer (Support Nodes). Support Nodes (SNs), which play the role of “supporting lemurs,” are intermediate servers that receive offloaded tasks from PNs and EDs. They manage execution queues using a hybrid Earliest Deadline First–Dynamic Resource Scheduling (EDF–DRS) policy, enforce feasibility constraints, and execute tasks using their local computational resources. After execution, SNs report status and results upward to the PNs and, when needed, back to EDs.

Cloud Layer (Back-End Analytics and Non-Critical Workloads). Above the fog layer, a cloud tier provides virtually elastic compute and storage resources. While LITO’s real-time offloading decisions primarily operate between EDs, SNs, and PNs, the cloud layer serves as a back-end for training and updating policy models using aggregated telemetry, executing delay-tolerant or batch workloads, and storing long-term logs. Conceptually, this completes the edge–fog–cloud continuum: latency- and energy-critical tasks are retained in the edge and fog strata, whereas non-urgent processing can be migrated to the cloud when beneficial.

System Coordination and Control Module. The system manager maintains a registry of all active devices and nodes, tracks their roles and capabilities, and enforces global configuration policies. It assists in node discovery, admission and departure handling, and the dissemination of updated offloading and scheduling policies across all SNs and EDs.

**Figure 2:**
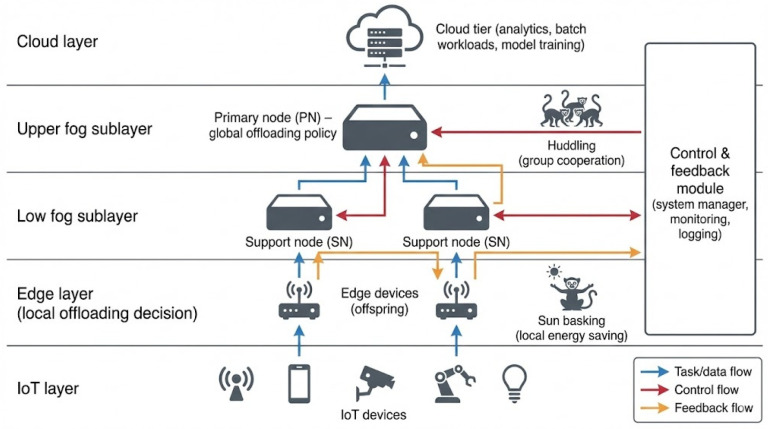
Lemur-inspired edge–fog–cloud infrastructure and node roles.

Feedback and Adaptation Module. This module gathers detailed performance metrics from EDs, SNs, and PNs, including throughput, energy consumption, task latency, and fairness indicators. It periodically aggregates these metrics and exposes them to the CSPL-CB learner and to off-line analytics in the cloud layer. This feedback loop enables continual adaptation of LITO’s decision policies to changes in workload patterns, node capacities, and network conditions. The overall multi-layered infrastructure is depicted in Figure 2.

### 3.4. Proposed Lemur-Inspired Task Offloading Algorithm

**Figure 3:**
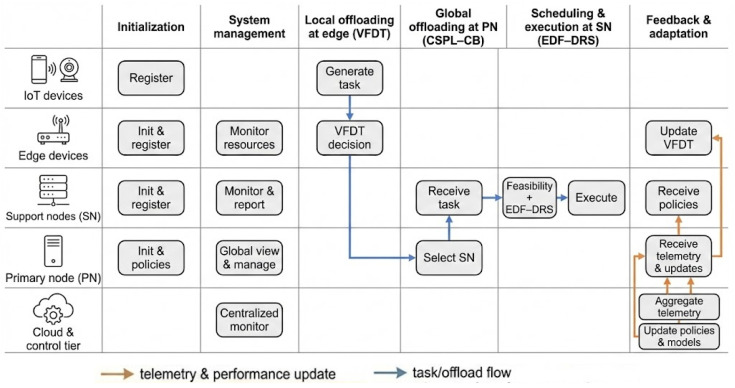
Role-based workflow of the LITO algorithm for decentralized task offloading.

**Figure 4:**
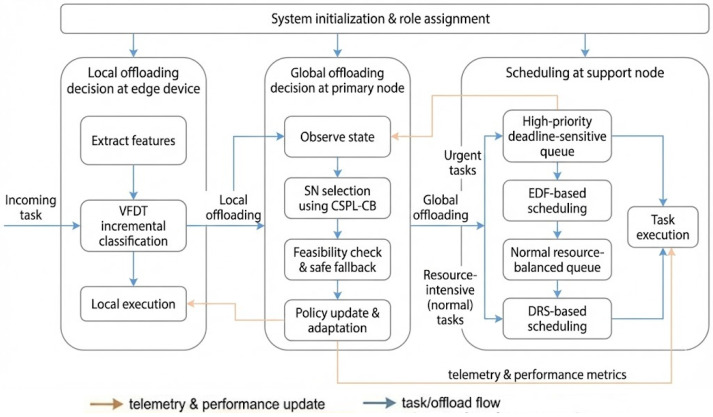
Component architecture of the LITO framework across the edge–fog–cloud continuum.

LITO is the algorithmic framework that instantiates the lemur-inspired behaviors described in Section 3.2 on top of the edge–fog–cloud infrastructure defined in Section 3.3. It specifies how IoT devices, edge devices, support nodes, and primary nodes cooperate to decide where tasks are executed and how resources are scheduled. The framework is organized into six main stages: system initialization, system management, local task offloading at edge devices, global offloading decisions at primary nodes, scheduling and execution at support nodes, and feedback and adaptation. Figure 3 presents the high-level, role-based workflow of these stages across the continuum tiers, while Figure 4 depicts the internal component architecture of the LITO framework.

Following initialization, each node carries out role-specific responsibilities within this workflow. IoT devices generate tasks and forward them to edge devices. Edge devices either execute tasks locally or, guided by Very Fast Decision Tree (VFDT) classifiers, offload them to primary nodes. Primary nodes transform global observations into offloading decisions using the CSPL-CB learner and distribute tasks to suitable support nodes. Support nodes then apply the hybrid EDF–DRS scheduler to execute tasks while balancing deadlines and load. Throughout this process, telemetry is collected and fed back to the coordination and learning components, enabling continual adaptation. Detailed algorithms for each stage are provided in Section 3.4.1, Section 3.4.2, Section 3.4.3, Section 3.4.4 and Section 3.4.5.

Following initialization, each node carries out role-specific resource monitoring, similar to behavioral sensing in lemur groups. EDs extract task-level features and use VFDT classifiers for real-time offloading decisions. PNs collect global telemetry from SN and apply CSPL-CB to refine offloading strategies. SNs implement a hybrid task scheduling mechanism that combines EDF for latency-sensitive tasks with DRS for load-balanced execution.

The LITO framework supports hierarchical task delegation. Tasks generated by IoT devices are first processed by EDs, which either execute them locally or offload them to PN based on VFDT outcomes. PNs aggregate telemetry, select suitable SNs based on CSPL-CB, and check whether execution on the chosen node is feasible. SNs then queue and execute tasks using EDF or DRS, depending on task priority and resource demand. The architecture incorporates two biologically inspired strategies: sun basking, which reflects proactive energy conservation at the edge layer by reducing local execution and preferring resource-rich nodes, and huddling, which represents coordinated task allocation from PNs to SNs on the basis of global offloading decisions for adaptive and balanced workload distribution within the fog layer. After task execution, all nodes submit performance feedback to the PN. This feedback loop allows continual policy refinement and helps maintain responsiveness and resilience under dynamic conditions. Figure 4 illustrates the system architecture of the LITO algorithm.

#### 3.4.1. Initialization

This phase coordinates all components in the network and ensures that each node operates according to its assigned role and responsibilities. It also supports the establishment of the fog computing environment, role assignment, network performance monitoring, and communication management across all entities in the system.

Initially, the system starts and loads with its configuration parameters to establish a connection with the centralized manager, retrieve essential setup data, and initialize system parameters based on predefined settings. This phase ensures that the network begins in a controlled and consistent state. After initialization, the processing power, storage capacity, and connectivity of each fog node are assessed to assign roles as PNs or SNs. EDs are designated as offspring nodes by default because of their resource constraints and reliance on fog nodes for task offloading. This structured role allocation mirrors the hierarchical organization observed in lemur groups and ensures that each node plays a role aligned with its capabilities. Algorithm 1 illustrates the initialization process.
**Algorithm 1:** Initialization**Input:** System configuration files, list of nodes, resource profiles of each node **Output:** Fully initialized fog system with assigned roles (PN, SN, ED) 1  Initialize the fog system by System Manager 2  Retrieve configuration data 3  **for** each fog node m_j_ **do** 4       Evaluate available resources 5       Assign role: PN (female lemur), SN (male lemur), or ED (offsprings) 6  **end** 7  Establish secure communication channels 8  Configure periodic monitoring and reporting: each SN and ED send its state vector (CPU, memory, queue length, energy, link quality) to the PN every ∆t time units

#### 3.4.2. System Management

Once roles are established, a system-wide monitoring solution is activated to continuously collect data on network traffic, node health, task execution, and resource utilization. This monitoring helps detect anomalies or performance bottlenecks early and supports stable and efficient operation. Secure communication channels are also established and maintained between the centralized system manager and all nodes so that the manager can distribute updated roles, configurations, and tasks, and nodes can report their status or negative feedback. This bidirectional communication keeps the network synchronized.

The system manager is responsible for role adjustments, global monitoring, and reporting. Based on information about node status, it can reassign roles when a node degrades or new resources are added. It also produces system-wide reports that summarize task completion rates, node health, and overall resource utilization, which can guide future optimization.

#### 3.4.3. Local Task Offloading in EDs Using VFDT

Local offloading enables EDs to handle their resources intelligently by deciding when to offload tasks to fog layer and when to execute them locally, while balancing local execution and remote processing. By offloading tasks appropriately, EDs can conserve energy, prolong operational lifetime, and improve application performance.

Existing ML classifiers, such as traditional Decision Trees, Random Forests, or Support Vector Machines, often require high memory and offline training and cannot efficiently manage streaming task data. This drawback leads to increased delay in dynamic environments. Moreover, they do not cope well with continuous adaptation to varying workloads or network conditions. Hence, VFDT (also called as Hoeffding Tree) is leveraged since it processes streaming data in real time, learns incrementally, and offers statistically confident decisions for dynamic task offloading with reduced computational cost. It also helps local task offloading to adapt continuously to changing workloads, network conditions, and device constraints within the fog environment. This adaptive mechanism is conceptually inspired by lemur behaviors such as sun basking and huddling, where lemurs regulate their energy and thermal balance (analogous to local offloading for energy optimization).

Initially, tasks are inspected to determine their suitability for offloading. Each task $t_{i}$ is analyzed in terms of CPU cycles, energy demand, memory requirement, and deadline. Tasks are then combined with metadata such as execution size, context, and priority. VFDT incrementally constructs a decision tree from the streaming task data, which makes it suitable for EDs where decisions must be made quickly and repeatedly.

When a $t_{i}$ instance arrives, its feature vector $X_{t}$ (as in Equation (1)) is fed to the VFDT to determine the class label: Offload (the task is offloaded to a PN) or Local Execution (the task is processed locally). The algorithm starts at the root node and moves down the tree by checking the attribute tests (splits) at internal nodes. At each ED $m_{j}$, VFDT keeps statistical summaries of the observed attributes at the leaf node. For numerical features, these statistics may include histograms, counts, or summary values essential for calculating split criteria during incremental learning. The decision-making process depends on Information Gain (G), which is given by:

(10) $$G\left(T,A\right)=H\left(T\right)-\sum_{v\in \mathrm{values}\left(A\right)}\frac{\left|T_{v}\right|}{\left|T\right|}H\left(T_{v}\right)$$

where H(T) indicates the task dataset T’s entropy at $m_{j}$; T_v_ indicates the subset split by attribute A; “values” (A) indicates the set of possible values or ranges that A can take. The Hoeffding Bound (ϵ) [43] is utilized to ensure statistically reliable splits. ϵ defines the confidence that the best attribute chosen after observing n instances is similar to the one that would be chosen with infinite data. ε is based on the range of the splitting criterion, the number of observed instances n, and the confidence parameter δ. The split criterion is applied:

If the best attribute $G_{best}$ is better than the second best $G_{2nd}$ by at least ϵ (i.e., $G_{best}$–$G_{2nd}$ > ϵ), split on the best attribute.

If $G_{best}$ and $G_{2nd}$ are within ϵ (i.e., $G_{best}$–$G_{2nd}$ ≤ ϵ), the standard VFDT would wait for more samples, but, here, a tie-break split is done to reduce decision latency.

**Figure 5:**
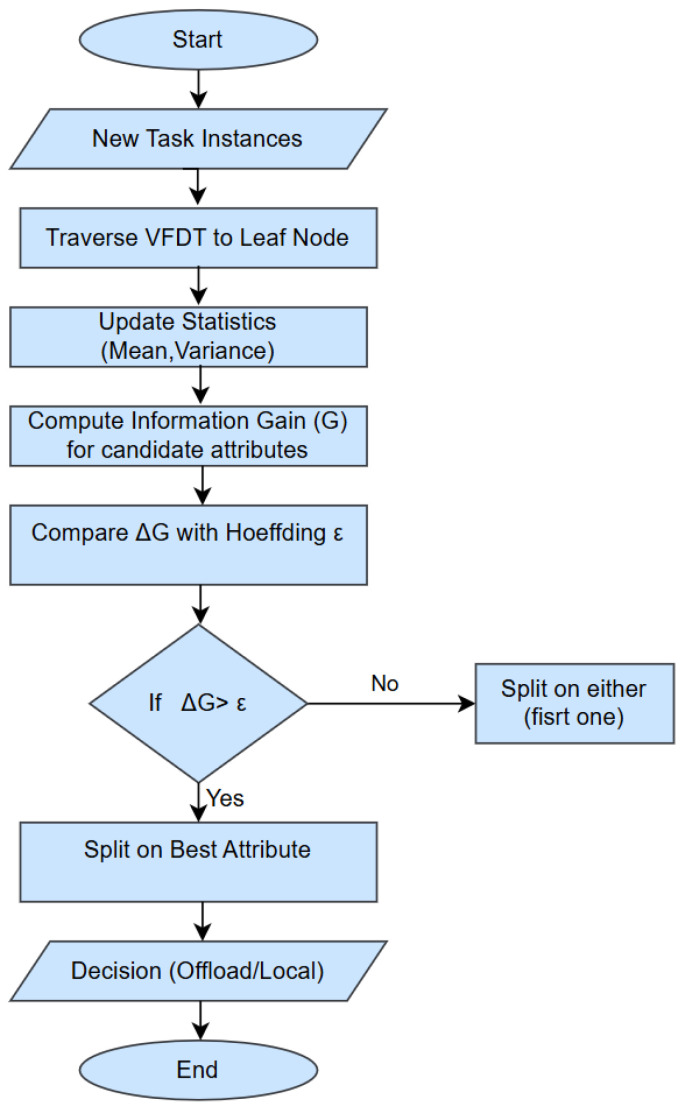
Local decision making using VFDT.

This split rule aids in avoiding premature decisions by ensuring that attributes with nearly equal gain are not selected incorrectly from limited samples. Using ϵ guarantees reliability with probability 1 − δ, whereas the selection of ϵ balances speed and accuracy. It ensures that VFDT makes confident splits only when sufficient samples have been gathered, reducing redundant complexity and enhancing computational efficiency. Until the task instance reaches a leaf node that does not have any splits, this process continues. Figure 5 illustrates the process of local decision making using VFDT.

When a decision is made to offload a task, the ED sends the packaged task data, metadata, and execution instructions to the PN over a secure, low-latency communication channel. Tasks that are classified as Local Execution typically require fewer CPU cycles, lower energy, and have short deadlines. These are executed locally to minimize communication overhead and response delay while conserving energy. Scheduling based on Earliest Deadline First (EDF) ensures that delay-sensitive tasks are executed quickly.

EDs also monitor offloaded tasks and manage responses from fog nodes. When the execution result is received, EDs integrate it into local applications. Each ED records performance metrics such as task completion time, network overhead, energy usage, and task success/failure rate, and feeds them back into the VFDT model. This mechanism allows the classifier to adapt over time to the dynamic fog computing environment. Since VFDT is an incremental learner, it improves its decision quality over time as more task instances are processed, without requiring retraining from scratch. Algorithm 2 describes the local offloading mechanism.
**Algorithm 2:** Local Offloading at EDs**Input:** Incoming task t_i_ **Output:** Local offloading decision: Local execution or offload 1  **for** each t_i_
**do** 2       Extract feature vector X_t_ 3       Feed X_t_ to VFDT 4       **while** traversing VFDT **do** 5         **if** Hoeffding Bound ϵ is satisfied **then** 6              Split the node 7         **end** 8       **end** 9       Classify t_i_: offload or Local Execution 10      **if** Decision = Local Execution **then** 11        Schedule the task using the EDF-based queue 12      **else** 13        Offload securely to PN for global decision-making. 14      **end**
15      Update VFDT incrementally with the observed outcome 16 **end** 17      Send periodic summary reports from each ED to the PN, including local queue length, execution latency, energy level, and offloading decisions

#### 3.4.4. Continual Supervised Offloading Policy with Contextual Bandit Feedback for PNs

PNs serve as the central intelligence of the fog computing network that aids in making global task offloading decisions while ensuring effective task distribution. Conventional RL methods such as Q-learning, Deep Q-Network, or the Actor-Critic method struggle in fog environments since they usually assume fixed state spaces, static task patterns, or require costly retraining when workload patterns evolve. In addition, they suffer from limited adaptability to non-stationary workloads and catastrophic forgetting. Hence, CSPL-CBis utilized, in which PN learns a policy online, preserving previously acquired knowledge. In contrast to RL-based methods, CSPL-CB does not depend on reward signals. Instead, it employs supervised oracle labels to update the offloading policy.

At each decision instant t, PN observes a compact state that summarizes network/ED/SN status and selects an offloading action that indicates which SN should receive each task. It then executes the decision by instructing SN, monitors outcomes, and updates its policy incrementally to adapt to evolving conditions. Similar to how lemurs regulate their group temperature through sun basking and huddling, the PN dynamically balances workload distribution by directing tasks toward underloaded or energy-efficient nodes while clustering tasks when latency or resource pressure rises. This adaptive mechanism reduces energy dissipation and promotes system stability under changing workloads.

In CSPL-CB, the environment is defined by a tuple (A, O, ρ), where A (or ΔY) is the discrete set of possible offloading actions (candidate SNs), O is the set of observations (X×Y) where the observation space contains context vectors $X_{t}$ and $Y_{t}$ is oracle labels available only at update time, and ρ( ) captures the non-stationary observation dynamics. At decision time t, PN receives the observations $O_{t}=\left(X_{t},Y_{t}\right)$ in which $X_{t}$ is given by,

(11) $$X_{t}=f(X_{\mathrm{mj}},X_{\mathrm{nls}},\;X_{\mathrm{ti}},\;X_{l})$$

$X_{t}$ aggregated vectors from SNs (X_mj_), such as available resources, utilization levels, queue lengths, and current acceptance rates. By utilizing exponentially weighted moving averages (EWMA) of latency and available bandwidth for each SN, network link statistics features ($X_{nls}$) are captured by the agent. Task-specific features (X_ti_) in Equation (1) and short local signal features (X_l_) like current PN load and an energy budget indicator are also added. f(⋅) is the function that performs normalization and compression to a fixed-size representation.

For each task $t_{i}$, PN predicts a probabilistic offloading distribution over available SNs: $a_{t}$ ∼ π$\left(\cdot |X_{t}\right)$, a_t_ ∈ A, where π indicates the stochastic policy. Entropy regularization is utilized during action sampling to maintain exploration. Prior to decision-making, each sampled action is fed through a feasibility filter to ensure that constraints like deadline feasibility, queue saturation limits, and minimum resource requirements are met.

(12) $$a_{t}^{F}=F(X_{t},a_{t})$$

If they are not met, a safe fallback is applied that assigns the task to the adjacent underloaded SN or queue task until the next offloading decision slot. This fallback can be compared to huddling behavior, in which nodes cluster computational tasks for mutual stability and minimal energy loss under constrained conditions, while ensuring overall system endurance. PN sends task assignment messages that include task metadata and deadlines to the chosen SN for execution. After execution, the PN collects an outcome tuple for each t_i_:

(13) $$O_{t}^{ti}=(L_{t}^{ti},E_{t}^{ti},S_{t}^{ti},{\Delta U}_{t}^{mj})$$

where $L_{t}^{ti}$ is the task latency at T; $S_{t}^{ti}$ ∈ {0,1} is the success indicator; $E_{t}^{ti}$ is the energy consumed by the task; ${\Delta U}_{t}^{mj}$ is the change in node utilization after task execution ($U_{t+1}^{mj}-U_{t}^{mj}$). The complete contextual feedback available to the PN at t is given by:

(14) $$O_{t}=\left\{O_{t}^{t_{i}}|t_{i}\in T\right\}$$

This contextual feedback, including energy, latency, success/failure signals, and utilization change, constitutes the bandit feedback required by CSPL-CB. The PN then utilizes this feedback to update its supervised policy in a continual fashion. This helps in enabling adaptive and constraint-aware task offloading under non-stationary workloads.

To enable supervised continual learning, the ground-truth SN label $Y_{t}$ is generated through a best-feasible oracle that assesses all candidate SNs under energy usage, deadline feasibility, and queue delay. The oracle chooses the SN minimizing a weighted cost function, and this offers the training signal:

(15) $$Y_{t}=arg\;\underset{a\in A}{min}\;C(a|X_{t})$$

This solves the log-loss definition by offering a clear, defensible correct action.

The policy is updated through the negative log-loss, which is given by:

(16) $$R_{t+1}=-logP_{\pi }(Y_{t}|X_{t})$$

which quantifies how well the agent predicts the oracle-optimal action. The long-run performance objective is given by:

(17) $$r^{\pi }=\underset{T\to \infty }{lim}inf\;E\left[\frac{1}{T}\sum_{t=0}^{T-1}R_{t+1}\right]$$

Maximizing $r^{\pi }$ obtains sustained accuracy in choosing near-optimal SNs under dynamic workloads.

To mitigate catastrophic forgetting under emerging workloads, the PN preserves a bounded rehearsal buffer of representative previous observations and periodically implements incremental updates with experience replay and regularization constraints. This mechanism enables the policy to combine new task patterns without ignoring historically valuable behaviors.

Since tasks are latency-critical, both inference and updates should satisfy decision latency per step $\leq \;L_{max}$, while ensuring the PN makes timely offloading decisions while continually optimizing its policy. By utilizing this continual supervised policy learning mechanism, the PN sustains deadline-aware, energy-efficient, and utilization-balanced performance across non-stationary fog environments. Algorithm 3 illustrates the global offloading approach.
**Algorithm 3:** Global Offloading Using CSPL-CB at PN**Input:** Offloaded tasks; current system state $X_{t}$; policy parameters **Output:** Selected SN; updated policy 1.  **for** each decision epoch t **do** 2.       Observe X_t_ from PN and SNs 3.       Sample action a_t_ 4.       **if** feasibility F(X_t_, a_t_) = false **then** 5.             Implement safe fallback 6.       **end** 7.       Perform action a_t_ and gather contextual bandit feedback $O_{t}^{ti}$ 8.        Generate a supervised label through the best-feasible oracle Y_t_ 9.        Calculate supervised loss L_t_ 10.         Store (X_t_, Y_t_) in replay buffer M 11.         Update policy incrementally using mini-batches from M 12.         **if** decision latency > L_max_ **then** 13.            Prune action space to satisfy real-time constraints 14.         **end** 15.   **end**

Coordination Between Local and Global Decision Layers: The coordination between local and global decision layers follows a hierarchical decision refinement mechanism. ED executes a binary decision (Local vs. Offload) through VFDT on the basis of local features $X_{i}$. When the decision is Local, execution remains at the ED without any global interaction. When the decision is Offload, task descriptor $X_{i}$ is sent to the PN together with local context metadata to perform multi-class global optimization using the CSPL-CB policy. Therefore, VFDT performs coarse-grained filtering, whereas CSPL-CB performs fine-grained routing amongst fog nodes. Decision conflicts are circumvented using mechanisms like role separation and feasibility filtering. EDs do not choose specific SNs; they only decide whether to offload. SN selection authority is preserved at the PN layer. Additionally, the PN applies the feasibility filter (Equation (12)) before final task assignment to enforce deadline, capacity, and utilization constraints. When the chosen SN becomes infeasible because of state changes, a safe fallback strategy allocates the task to the next best possible SN. This hierarchical authority structure guarantees global constraint compliance by avoiding contradictory routing decisions. After the execution of tasks, outcome feedback $\left(L_{t}^{t_{i}},E_{t}^{t_{i}},S_{t}^{t_{i}},\Delta U\right)$ is sent back to both PN and ED. The PN updates the CSPL-CB policy, whereas the ED updates its VFDT model by utilizing the execution results. This dual-feedback mechanism confirms that local offloading decisions gradually align with global optimization trends by minimizing long-term decision divergence.

#### 3.4.5. Scheduling Offloaded Tasks in SNs

Once the task is sent to SN from PN, SN takes full responsibility for its local scheduling and execution. Scheduling is performed with the help of a hybrid EDF-DRS (Earliest Deadline First-Dynamic Resource Scheduling) scheduler, tasks depending on resource availability, priority, and execution efficiency. This scheduler analyses resource requirements of tasks, prioritizes tasks based on task size, deadlines, or dependencies, and orders the execution to meet deadlines. In this hybrid approach, two logical queues, such as a high-priority deadline-sensitive queue and a normal resource-balanced queue, are utilized. This two-queue split allows SN to balance strict timing guarantees and adaptive resource distribution.

Initially, tasks are analyzed for their resource requirements and deadlines. Tasks with strict deadlines are kept in a high-priority deadline-sensitive queue (Q_EDF_) that uses EDF scheduling. In contrast, tasks that are resource-intensive or have relaxed timing requirements are kept in the normal resource-balanced queue (Q_DRS_) that uses DRS scheduling [44]. Q_EDF_ manages urgent tasks and is frequently resorted to maintain deadline order, while Q_DRS_ manages less time-critical tasks and is resorted to only periodically to conserve computation. Like how lemurs dynamically switch between sun basking for conserving energy and huddling to maintain warmth and group balance, the hybrid scheduler switches between Q_EDF_ and Q_DRS_ to maintain thermal-equilibrium-like stability in resource allocation, prioritizing urgent processes when needed. It preserves energy efficiency during low-load conditions. This queue separation aids in minimizing redundant resorting, ensuring that the hybrid scheduler quickly prioritizes urgent tasks with reduced overhead.

For each $t_{i}$ with deadline $D_{t_{i}}$ and remaining workload $W_{t_{i}}$ at time t, its urgency factor (${UF}_{t_{i}}$) is given by:

(18) $${UF}_{t_{i}}=\frac{W_{t_{i}}}{D_{t_{i}}-t}$$

Ensure that $D_{t_{i}}-t$ > 0. If ${UF}_{t_{i}}$ exceeds a predefined threshold, the task is kept in Q_EDF_; otherwise, it is kept in Q_DRS_. Q_EDF_ is always given priority in execution. Tasks in this queue are scheduled in increasing order of their deadlines to ensure that SN minimizes the risk of missed deadlines. For any two tasks $t_{i}$ and $t_{j}$ in Q_EDF_, the ordering follows: $t_{i}$ < $t_{j}$ when $D_{t_{i}}$ < $D_{t_{j}}$. By considering available CPU, memory, and bandwidth at each scheduling interval, Q_DRS_ operates adaptively. Each task in Q_DRS_ is assigned a proportional share of resources depending on its requirements. When the available computational capacity of m_j_ is C_f_, and a task t_k_ requires R_k_ units of computing, its assigned share S_k_ is determined by:

(19) $$S_{k}=\frac{R_{k}}{\sum_{j\in Q_{DRS}}R_{j}}\times C_{f}$$

This proportional allocation guarantees fairness among non-urgent tasks, preventing resource starvation. This mechanism reflects lemur huddling behavior in which shared heat represents equitable distribution of limited energy among members, while ensuring that no individual suffers a resource deficit. Task execution proceeds by dispatching all possible Q_EDF_ tasks in order of deadline, using the required portion of the available computational resources. Based on the computed shares, the remaining computational capacity is then distributed amongst tasks in Q_DRS_. When the EDF set surpasses the free capacity of SN, the scheduler leverages proportional reduction within Q_EDF_, providing higher weight to tasks with the earliest deadlines. This hybrid method ensures that critical tasks are prioritized while making effective utilization of available resources for normal tasks.

During execution, SN continuously monitors task completion time, actual resource consumption, and deviations from estimates. When tasks from Q_DRS_ start to lag excessively, SN can temporarily elevate them into Q_EDF_ by maximizing their ${UF}_{t_{i}}$. On the other hand, DRS can expand allocations to accelerate heavier tasks when resources are underutilized. This behavior is similar to changing from huddling to sun basking when environmental energy levels allow higher activity for thermal and computational balance. After execution, task latency, success indicator, energy consumed and change in node utilization are reported back to the PN, enabling better coordination in future offloading decisions. Algorithm 4 shows the offloading process in SNs.
**Algorithm 4:** Scheduling of Offloaded Tasks in SNs**Input:** Tasks assigned by PN; Urgency factors UF_ti_ **Output:** Ordered execution of tasks; balanced resource distribution; outcome tuples 1   **for** each t_i_ at SN **do** 2         Calculate UF_ti_ 3         **if** UF_ti_ > threshold **then** 4           Place t_i_ in Q_EDF_ 5         **else** 6           Place t_i_ in Q_DRS_ 7         **end** 8   **end** 9 **end**
***// EDF Queue Management//*** 10    Sort Q_EDF_ based on deadlines 11    **while** Q_EDF_ ≠ ∅ **do** 12       Execute the earliest-deadline task while coordinating nodes through adaptive load balancing inspired by lemur huddling behaviour 13    **end** ***// DRS Queue Management//*** 14    **if** the reschedule interval is reached **then** 15       Resort Q_DRS_ 16    **end** 17    **for** each task t_k_ ∈ Q_DRS_
**do** 18       Assign proportional share S_k_, considering workload equilibrium for sustaining node efficiency 19    **end** 20    **if** combined demand of Q_EDF_ > available C_f_
**then** 21       Proportionally reduce allocations across tasks using S_k_ 22    end 23    Report outcome tuples back to PN

## 4. Evaluation Methodology

To assess the viability of the proposed LITO algorithm, the iFogSim toolkit [46], an extension of CloudSim [47], is employed, which is a widely utilized open-source Java-based toolkit developed for fog computing simulations. It depends on a distributed dataflow overview and functions based on the Sense–Process–Actuate paradigm. iFogSim allows event-driven interactions amongst edge and fog components, enabling the modeling of real-time computation offloading scenarios. It assumes distributed task generation and processing across heterogeneous IoT and fog nodes. This framework offers vital classes like Sensor, EdgeDevice, Actuator, Tuple, PhysicalTopology, and Application, which together enable the development of customized edge/fog computing environments with several heterogeneous nodes.

Workloads are synthetically generated with probabilistic distributions for memory, CPU cycles, and arrival times, allowing controlled exploration of light to high-intensity task scenarios. The network model integrates realistic bandwidth and latency parameters for IoT-to-Edge and Edge-to-Fog links, by capturing transmission delays under diverse data rates. Parameter sensitivity is analyzed systematically by changing task complexity, workload size, data rates, and node topologies, enabling evaluation of robustness, SLA adherence, and performance of LITO under both nominal and stress conditions.

### 4.1. Simulated Scenarios

To evaluate the scalability and responsiveness of the LITO algorithm under varying edge workloads, we define four simulation scenarios as shown in Table 3. Each scenario increases the number of IoT devices and associated Edge Devices, while maintaining a constant fog-layer structure (PNs, SNs, and System Manager). Fog nodes remain constant since the focus is on assessing offloading efficiency of LITO under increasing edge workloads, instead of scaling fog infrastructure. This incremental topology supports systematic analysis of LITO’s behaviour across small- to large-scale deployments. The scenarios are defined as follows:

- Lightweight Edge Load (Configuration 1): Models a small-scale environment with 16 IoT devices and 8 EDs, representing use cases such as room-level smart automation.
- Moderate Edge Load (Configuration 2): Doubles the edge scale to 32 IoT devices and 16 EDs, representing a smart building or floor.
- Dense Edge Load (Configuration 3): Includes 64 IoT devices and 32 EDs, approximating a complex system like a smart industrial floor or campus.
- High-Density Edge Load (Configuration 4): Stress-tests LITO with 128 IoT devices and 64 EDs, simulating dense city blocks or wide-area fog deployments.

**Figure 6:**
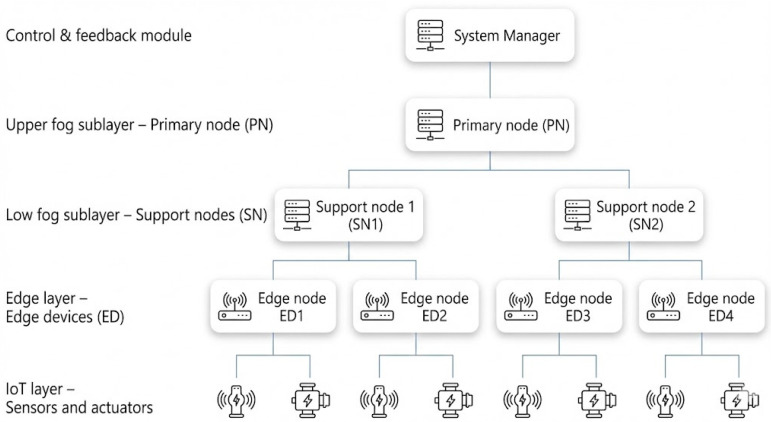
Topology illustrating hierarchical interactions among system devices.

In all configurations, the fog layer includes 1 PN, 3 SNs, and 1 System Manager, which remain constant across different scenarios. This configuration spectrum enables detailed evaluation of how LITO handles increasing device density and varying levels of workload intensity. The corresponding topology is illustrated in Figure 6. Simulation configurations detailing the number of IoT devices, EDs, PNs, SNs, and system managers are illustrated in Table 3.

**Table 3:** Simulation configurations detailing the number of IoT devices, EDs, PNs, SNs, and system managers.

Parameter/Category	IoT Devices	EDs (Offspring Nodes)	Fog Devices (SN + PN)	System Manager
Tuple Type	Sensor: ENV_SENSOR Actuator: DISPLAY	—	—	—
Distribution	Sensor: Based on workload size	—	—	—
Latency	Sensor: 1.0 ms (to gateway/edge) Actuator: 1.0 ms (response time to user)	To Parent (SN): 2 ms (Uplink)	Support → Primary: 5 ms Primary → Registry: 10 ms (Uplink)	System Manager → Nodes: 0–10 ms (Downlink)
Node Name	—	Edge	SN: support- PN: primary-	System Manager
MIPS	—	10,000	SN: 30,000 PN: 50,000	20,000
RAM	—	8000 MB	SN: 20,000 MB PN: 40,000 MB	16,000
Uplink/Downlink Bandwidth	—	4000 Mbps	SN: 8000 Mbps PN: 10,000 Mbps	5000 Mbps
Level	4	3	SN: 2 PN: 1	0
Rate per MIPS	0	0.01	0.01	0
Busy Power	2 W	100 W	SN: 150 W PN: 200 W	120 W
Idle Power	1 W	60 W	SN: 90 W PN: 100 W	80 W
CPU usage cost (G$/s)	0.05	0.08	SN: 0.22; PN: 0.35	0.3
Memory usage cost (G$/MB)	0.005	0.008	SN: 0.022; PN: 0.035	0.03
Bandwidth usage cost (G$/MB)	0.01	0.015	SN: 0.025; PN: 0.03	0.02
Modules Deployed	task_generator	—	primary_planner; support_scheduler	system_manager
Functional Role	Task generators	Local executors	PN: Global controllers SN: Mid-layer schedulers	System manager

The hierarchical topology utilized in all simulation scenarios is summarized as follows:

- IoT Layer: variable, ranging from 16 to 128 IoT devices based on the configuration.
- Edge Layer: variable, ranging from 8 to 64 EDs.
- Fog Layer: Fixed, with 1 PN, 3 SNs, and 1 system manager across all configurations to isolate the impact of increasing device density and edge workload on the performance of LITO.

The fog layer is actively involved in task execution and coordination across all configurations. PNs act as global planners to make offloading decisions using the CSPL-CB mechanism, but do not directly complete tasks. All tasks offloaded from EDs are sent to SNs, acting as the actual execution entities in the fog layer. Therefore, PNs execute policy refinement and coordination, whereas SNs manage scheduling and computation of offloaded tasks. This separation of execution and planning roles remains consistent across all scenarios.

In the experiments, the weighting coefficients in Equation (2) are fixed as α = 0.5, β = 0.3, and γ = 0.2 to prioritize latency, considering energy consumption and utilization imbalance. These values are chosen to ensure balanced trade-offs amongst conflicting objectives and reflect latency-sensitive fog applications. The weights are normalized so that α + β + γ = 1.

In Table 3, the simulation parameters are chosen to emulate a realistic hierarchical fog computing environment with gradually increasing computational capacity, bandwidth, and energy consumption from EDs to PNs. The latency values (1–10 ms) show proximity-based communication delays reported in MEC and fog simulation studies. In contrast, MIPS and RAM configurations follow a tiered scaling pattern, indicating constrained EDs and high-capacity fog nodes. Power and cost coefficients are proportionally allocated to capture realistic differences in energy consumption and pricing across different layers. The proposed framework’s robustness is assessed under changing dynamic task arrivals and workload intensities, while ensuring that performance trends are not based on a single static configuration.

### 4.2. Synthetic Workload Generation

To evaluate the LITO framework under a wide range of conditions, the experiments employ a fully synthetic workload generator designed to model realistic, heterogeneous resource demands and dynamic task arrivals across a multi-layer IoT–fog architecture. While real data center traces offer valuable insights, they often lack the flexibility and granularity required to systematically test decentralized offloading behaviors across diverse deployment scenarios. In contrast, a synthetic approach allows controlled generation of workloads that reflect a broad spectrum of edge and fog environments, including underexplored or future-oriented configurations. Inspired by the Google Borg cluster workload traces (ClusterData2019/trace version 3; May 2019) [48], the synthetic workload is generated to reflect realistic large-scale computing patterns. Each task, node, and network state is modeled with key compute, memory, I/O, timing, bandwidth, latency, and execution outcome attributes. The simulation environment a hierarchical IoT–edge–fog topology whose computational capabilities are parameterized on the basis of typical edge and fog hardware specifications. The number of active nodes in each layer is given in Section 4.1. Tasks originating from IoT devices are either processed locally on EDs or offloaded to fog nodes based on network conditions and resource availability. Bandwidth, network latency, and energy consumption are simulated for IoT → Edge → Fog links. Performance is assessed in terms of vital metrics like task latency, energy consumption, makespan, resource utilization, throughput, success rate, and offloading ratio, thereby providing a controlled yet realistic environment to test the efficiency of the proposed framework.

Each simulation scenario was repeated 10 times using different random seeds. Service times were generated from the specified task-complexity distributions, while task arrivals followed a Poisson process with exponentially distributed inter-arrival times. Reported results are means across runs; error bars indicate ±1 standard deviation over the 10 runs.

### 4.3. Experimental Variables

The experiments were designed to investigate the impact of the following main factors:

- Workload Size (Tasks per Second): Tested at 100, 200, 300, and 600 tasks per second to simulate different levels of sensor activity and user demand.
- Task Complexity (CPU cycles): Measured at 10, 20, 30, 40, and 50 × 10^6^ CPU cycles, representing a range of task types, from lightweight sensor processing to compute-intensive video analytics or anomaly detection.
- Network Bandwidth (Mbps): Evaluated at 5, 10, 20, 50, 100 Mbps to test the system
- Node Configuration (System Topology): Four hierarchical topologies (Configurations 1 to 4) were designed, each corresponding to one of the simulation scenarios in Table 3. Workload size varies from 60 to 900 tasks per second across different configurations (Table 4). To measure TSR, workload size varies from 60 to 90 tasks per second across different configurations.

Low workload sizes (60–90 tasks/s) were utilized to measure TSR in order to ensure that the system is not saturated and to accurately capture task completion behavior under controlled conditions. On the other hand, overall experiments listed in Table 4 varied workload from 60 to 900 tasks/s (Table 4) to evaluate performance under heavier system loads.

### 4.4. Experimental Design

**Figure 7:**
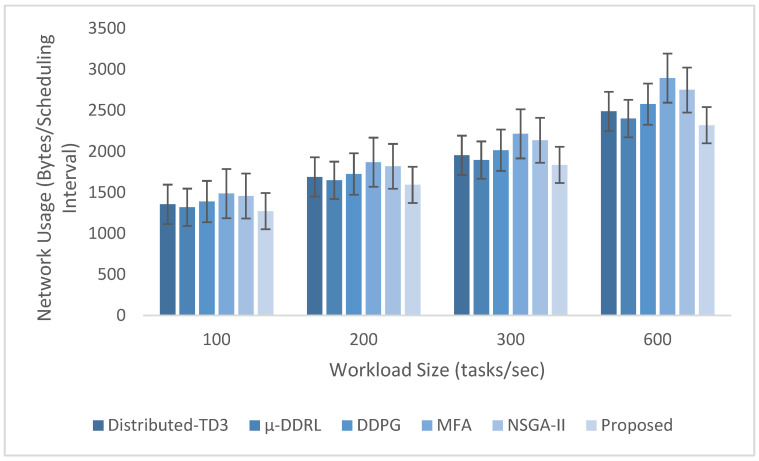
Results of average network usage (bytes per scheduling interval) across varying workload sizes.

**Figure 8:**
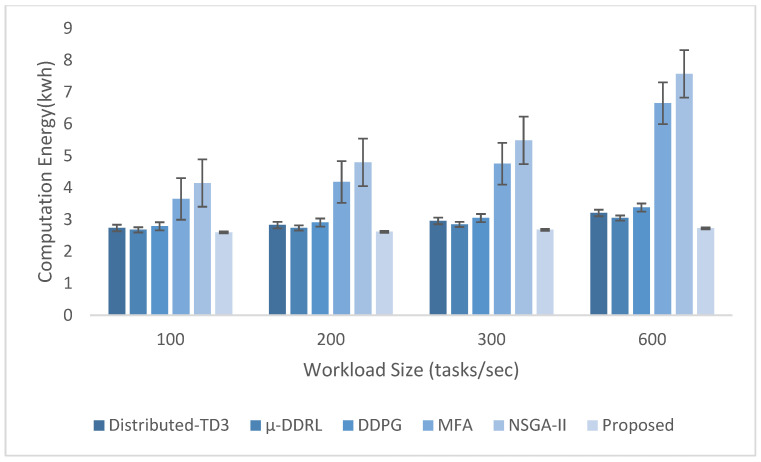
Comparison of the computation energy consumption (kWh) across varying workload sizes.

**Figure 9:**
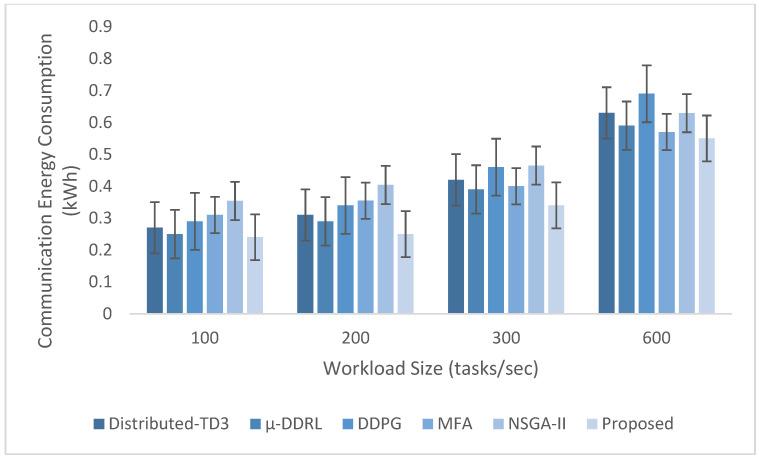
Comparison of the communication energy consumption (kWh) across varying workload sizes.

**Figure 10:**
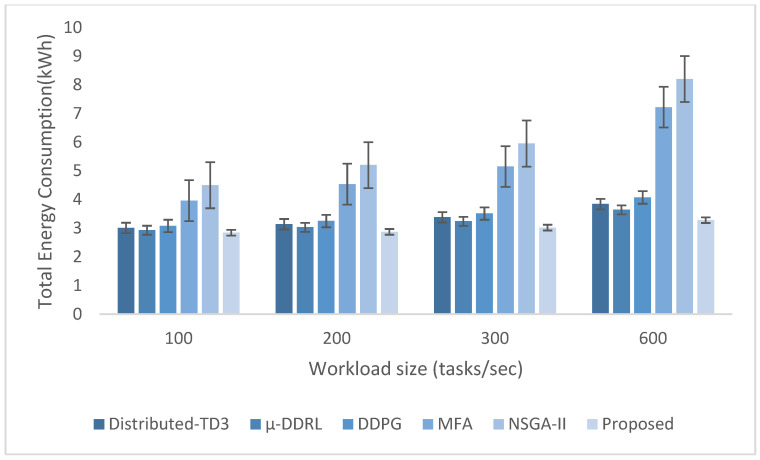
Comparison of the total energy consumption (kWh) across varying workload sizes.

**Figure 11:**
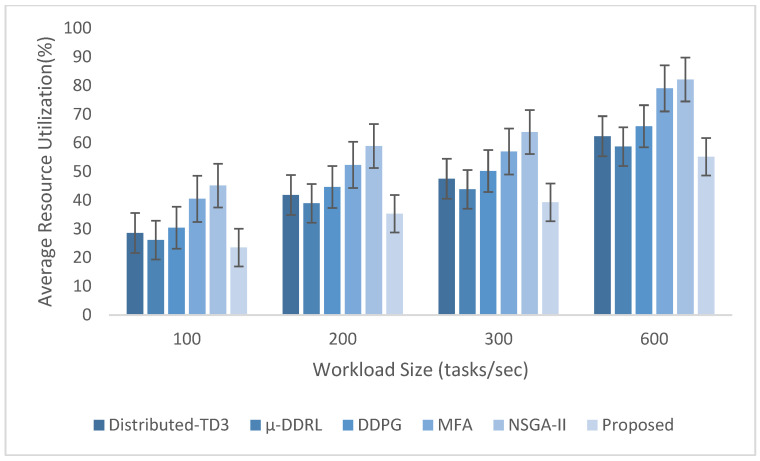
Comparison of the average resource utilization (%) across varying workload sizes.

**Figure 12:**
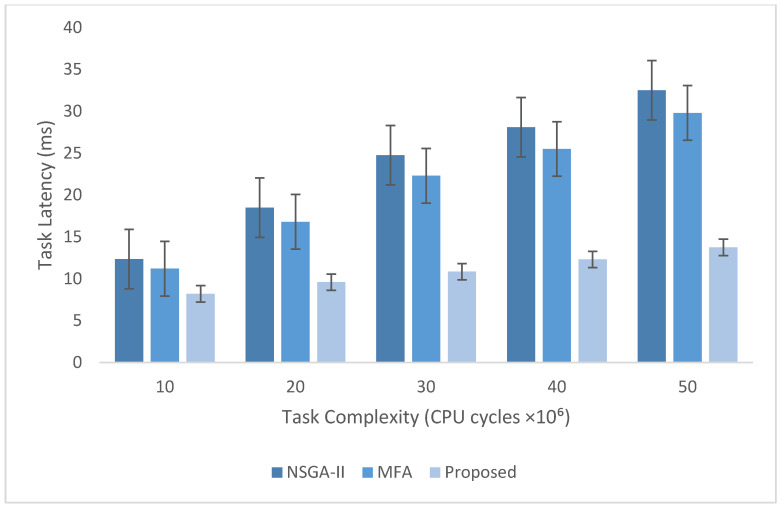
Comparison of the task latency across varying task complexities.

**Figure 13:**
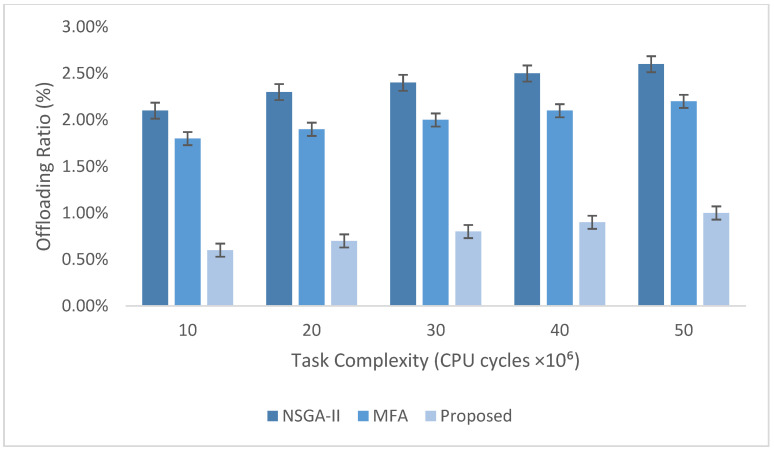
Comparison of the offloading ratio across varying task complexities.

The experimental evaluation was organized as a set of targeted experiments, each modeled to isolate the influence of a specific system factor on LITO’s performance under controlled conditions. The experiments were organized as follows:

- Experiment E1: Impact of Workload Size (Lightweight Edge Load): It assesses the system’s behavior under increasing workload intensity. The lightweight topology was fixed, whereas the workload size was varied across {100, 200, 300, 600} tasks per second. The data rate was set to 20 Mbps, while task complexity was fixed at 20 × 10^6^ CPU cycles. Performance was evaluated with the metrics such as network usage, communication energy, computation energy, total energy consumption, and average resource utilization (Figure 7, Figure 8, Figure 9, Figure 10 and Figure 11).
- Experiment E2: Impact of Task Complexity (Lightweight Edge Load): For examining sensitivity to computational demand, task complexity was varied from 10 × 10^6^ to 50 × 10^6^ CPU cycles, whereas the workload size was fixed at 100 tasks per second under Lightweight Edge Load. Moreover, the data rate was set at its default value. It evaluates task latency and offloading ratio (Figure 12 and Figure 13).
- Experiment E3: Impact of Workload Characteristics and Topology: It examines the impact of arrival dynamics and network conditions. Task Success Ratio (TSR), throughput, makespan, computational cost, task completion time, task success ratio, offloading ratio, and SLA violation rate were calculated against varying workload sizes across four deployment configurations given in Section 4.1. Task latency was assessed under diverse data rates {5, 10, 20, 50, 100} Mbps, with workload size fixed at 100 tasks/s under Lightweight Edge Load.
- Experiment E4: Ablation Study on Execution Scenarios: An ablation analysis was performed to evaluate the contribution of different execution strategies, such as ED-only local execution, full offloading, hybrid execution at the edge, SN-only execution, and hybrid SN + ED execution. All the System parameters and workload conditions were held constant, and performance was assessed in terms of throughput and makespan.

This experiment-centric design aligns the evaluation methodology with the reported results. To account for stochasticity in the simulator and workload generation, each scenario (algorithm × workload level) was executed 10 independent times using different random seeds. Reported results correspond to the mean across runs. In figures that include error bars, the bars represent ±1 standard deviation computed over the 10 runs.

### 4.5. Performance Metrics

The performance metrics considered are:

- Network Usage: It is the average number of bytes sent per scheduling interval during the simulation. It comprises task descriptors, control messages, and data exchanged during task offloading between IoT devices, EDs, and fog nodes. The metric is normalized per fixed time window to enable fair comparison across different workload sizes.
  (20) $$Network\;Usage=\frac{1}{T_{sim}}\sum_{k=1}^{K}B_{k}$$
  where $B_{k}$ indicates the total bytes sent during interval k; $T_{sim}$ indicates the total simulation time, and K indicates the number of scheduling intervals.
- Makespan: It is the total time taken to complete all submitted IoT tasks across the multi-layer LITO overview. Reducing makespan is vital for efficient scheduling and faster task completion under changing workloads.
  (21) $$Makespan=\underset{i\in T}{\max}\;C_{i}$$
  where $C_{i}$ is the completion time of task *i*.
- Average Resource Utilization: It evaluates how efficiently computational and communication resources of devices are exploited during task execution. It assesses the balance between idle capacity and workload distribution across fog/edge layers.
  (22) $$Average\;Resource\;Utilization=\frac{\sum_{i=1}^{N}{TVM}_{i}}{Makespan\times N}$$
  where ${TVM}_{i}$ is the total time taken by the ith virtual machine to complete all allocated tasks, and N is the total number of resources.
- Task Latency: It is the overall time taken from task generation at an IoT device until its execution is completed and the output is returned. It shows the end-to-end delay caused by a task in the system.
  (23) $$L_{i}=L_{i}^{Q}+L_{i}^{T}+L_{i}^{E}+L_{i}^{R}$$
  where $L_{i}^{Q}$ is the queuing delay before scheduling or offloading the task; $L_{i}^{T}$ is the transmission delay caused when sending the task data from an IoT device to the nearby ED; $L_{i}^{E}$ is the processing time of the task at the allocated node; $L_{i}^{R}$ is the return time.
- Computation Energy Consumption: It indicates the total energy used by processing entities like IoT devices, EDs, and fog nodes during the execution of computational tasks.
  (24) $$E_{comp}=\sum_{j\in M}\left(E_{j}^{busy}\cdot T_{j}^{exec}+E_{j}^{idle}\cdot T_{j}^{idle}\right)$$
  where M indicates the set of processing nodes; $P_{j}^{idle}$ indicates the energy consumption of node j in idle state (W); $P_{j}^{busy}$ indicates the energy consumption in the busy state (W); $T_{j}^{exec}$ indicates the total execution time at j; $T_{j}^{idle}$ indicates the idle time of j.
- Communication Energy Consumption: It indicates the total energy required to send tasks and associated data among IoT devices, EDs, and fog nodes during task offloading.
  (25) $$E_{comm}=\sum_{(i,j)\in L}E_{ij}^{tx}\cdot T_{ij}^{tx}$$
  where L indicates the set of communication links; $P_{ij}^{tx}$ indicates the transmission power over the link $\left(i,j\right);\;T_{ij}^{tx}$ indicates the transmission time over the link $\left(i,j\right)$.
- Total Energy Consumption: It is the total energy utilized by all entities during the complete life cycle of task execution that includes computation and communication activities.
  (26) $$E=\sum_{i=1}^{N}E_{i}^{Comp}+E_{i}^{Comm}$$
  where $E_{i}^{Comp}$ is the computation energy utilized for executing task i on IoT device, ED, or fog node $E_{i}^{Comp}$ is the communication energy needed for sending task i among IoT devices, EDs, and fog nodes.
- Throughput: It calculates the rate at which tasks are effectively completed within a given time period, reflecting the processing efficiency of the proposed system.
  (27) $$Throughput=\frac{N_{completed}}{T_{sim}}$$
  where $N_{completed}$ indicates the number of successfully completed tasks; $T_{sim}$ indicates the overall simulation time. Throughput is expressed in tasks per unit time.
- Computational Cost: It is the total expenditure of system resources, such as CPU, memory, and bandwidth, incurred during the communication and execution of all tasks. It is expressed in Grid Dollars (G$).
  (28) $$CC=\sum_{i=1}^{N}{CC}_{i}^{cpu}+{CC}_{i}^{m}+{CC}_{i}^{bw}$$
  where ${CC}_{i}^{cpu}$ is the CPU usage cost; ${CC}_{i}^{m}$ is the memory usage cost; ${CC}_{i}^{bw}$ is the bandwidth usage cost.
- Offloading Ratio: The offloading ratio measures the ratio of tasks sent from EDs to higher-tier fog nodes for processing, relative to the total tasks generated.
  (29) $$Offloading\;ratio\;(\%)=\frac{N_{offloaded}}{N_{total}}*100$$
- SLA Violation Rate (%): It directly evaluates the real-world acceptability of how frequently deadlines/latency bounds are missed.
  (30) $$SVR(\%)=\frac{N_{violated}}{N_{total}}\times 100$$
  where $N_{violated}$ indicates the number of tasks missing the deadline/latency constraint.
- Task completion time (TCT): It measures the average time needed for a task to be completed:
  (31) $$TCT=\frac{1}{N}\sum_{i=1}^{N}T_{i,k}$$
  where $T_{i,k}$ is the execution time of task *Ti* at computing node *k*. When TCT is low, task execution efficiency is improved.
- Task success ratio (TSR): It estimates the proportion of tasks completed before their deadlines:
  (32) $$TSR=\frac{\sum_{i=1}^{N}I(T_{i,k}\leq D_{i})}{N}$$
  where *D*_*j*_ indicates the deadline; I() indicates an indicator function. A higher TSR shows a more reliable system.

In this study, we compare LITO with two established multi-objective metaheuristics adapted to the task-offloading formulation, Non-dominated Sorting Genetic Algorithm II (NSGA-II) [39] and the Multi-Objectives Firefly Algorithm (MFA) [36], and three representative distributed DRL–based offloading approaches, including Distributed-TD3 [42], µ-DDRL [43], and DDPG [44]. These baselines were chosen according to three criteria. First, both algorithms natively support multi-objective or composite optimization. They can be configured with the same objective vector (energy, latency, and resource-utilization balance) and constraints as LITO, which enables a fair comparison at the problem level. Second, these baselines have both been used in prior work on resource allocation and task offloading in cloud/edge/fog environments. They are widely regarded as representative state-of-the-art evolutionary, swarm-based, and distributed learning-based optimization (DRL) approaches, respectively. Third, they belong to two different algorithmic families (evolutionary and swarm-intelligence), so that the performance gains achieved by LITO are not tied to the weaknesses of a single optimization paradigm. The inclusion of distributed RL frameworks confirms that LITO is assessed against adaptive, online decision-making strategies instead of only static/population-based optimizers.

We deliberately avoid adding a large number of closely related metaheuristics (such as further NSGA-II NSGA-II variants or minor DRL modifications), since these show similar convergence behavior under similar objective settings and would not materially modify the comparative insights. Instead, we complement the comparison with external baselines by including an ablation study that isolates the effect of LITO’s design choices (local execution only, full offloading, hybrid ED policy, SN-only execution, and the proposed SN + ED collaboration). This combination of strong external baselines and internal ablations provides a focused yet informative evaluation of the proposed framework.

Each baseline is simulated under similar workload distributions, network topologies, and system constraints, ensuring fair comparison. For baseline MFA and NSGA-II, the objectives are reducing task latency and energy consumption, with MFA also maximizing resource utilization. For distributed RL methods, the objective functions integrate latency–energy trade-offs using reward formulations within actor–critic frameworks for online adaptation to workload variations. Decision variables include task-to-node offloading assignments to fog or cloud nodes, subject to constraints on task deadlines, node capacity, cluster size, as well as network bandwidth. Baseline algorithms are implemented in iFogSim using hierarchical fog-cloud topologies by modeling tasks, dependencies, and control loops through AppModule, AppEdge, and AppLoop classes. Balanced resource allocation and fairness are ensured via cluster-head selection, load balancing, and equitable task scheduling across devices. Performance metrics are assessed through repeated simulations. Averaged results are utilized for comparative analysis.

## 5. Experimental Results

This section provides the results after the evaluation of the proposed LITO algorithm by analyzing its performance under diverse workload sizes, workload characteristics, task complexities, and system configurations. The aim was to analyze how different factors influence the performance metrics related to energy efficiency, latency, resource utilization, and offloading behavior in fog computing environments.

### 5.1. Impact of Workload Size

Under an increasing workload, LITO maintains controlled growth in network usage without sudden spikes or early saturation, indicating resilient scaling. As shown in Figure 7, network usage increases gradually with workload, reflecting a proportional rise in task offloading and data exchange among IoT devices, EDs, and SNs/PNs. This contrasts with baseline methods, where aggressive offloading under high load can trigger network bursts and congestion. LITO achieves smoother behavior by balancing local execution and offloading decisions through role-based ED–SN–PN coordination. In particular, feasibility filtering prevents forwarding infeasible tasks, reducing unnecessary transmissions and retransmissions. In addition, the huddling-inspired mechanism enables SNs and PNs to share load under stress, which mitigates localized overload and avoids communication bursts. Overall, the gradual increase in normalized network usage suggests that LITO scales effectively with workload while keeping communication overhead manageable. Practically, this supports admitting larger task volumes without risking network saturation, which is critical in bandwidth-limited fog deployments.

Figure 8, Figure 9, Figure 10 and Figure 11 show the comprehensive evaluation of the proposed and existing task offloading strategies in terms of computation energy, communication energy, total energy consumption, and average resource utilization under increasing workload intensity. Distributed RL methods also demonstrate adaptive computation reduction when compared to metaheuristic baselines; however, their continuous policy updates and neural network inference lead to additional processing overhead under high workload conditions. Across all workload sizes under Lightweight Edge Load, the proposed LITO framework reliably proves lower computation energy and higher resource efficiency when compared with existing NSGA-II and MFA frameworks, as shown in Figure 8. It indicates that tasks are more capably distributed and processed across EDs and fog nodes. The improvement arises since LITO combines feasibility-aware global offloading with EDF-DRS based local scheduling, minimizing idle cycles and preventing costly re-executions due to deadline violations. In addition, the role-based selection mechanism restricts oscillatory offloading between nodes, lowering processing churn, while cooperative huddling spreads workload among neighbor nodes during transient spikes. These effects together translate into reduced local processing overhead, similar to how lemurs balance metabolic expenditure via coordinated sun basking behaviors. Practically, this indicates that operators can assist higher request volumes without scaling calculate resources or breaching energy budgets.

Under increasing task load, LITO attains reduced communication energy consumption when compared to baselines. Even though distributed RL methods like µ-DDRL and Distributed-TD3 minimize centralized bottlenecks, they have increased communication overhead because of state exchange, policy dissemination, and experience synchronization between agents. As in Figure 9, baselines show increasing energy costs when workload increases, while LITO remains consistently lower even in the high-load region. This is mainly due to feasibility filtering and role-based offloading (ED/SN/PN), which avoid wasted transmissions and oscillatory task migration. Practically, this produces improved energy-efficient task handling in dense edge deployments.

The total energy consumption shown in Figure 10 consolidates these improvements, highlighting that LITO outperforms both benchmark strategies in overall system efficiency. LITO exploits CPU, memory, and bandwidth evenly across the hierarchy, in contrast to MFA which remains limited by clustering heuristics or NSGA-II which over-utilizes hotspots at high loads. The RL-based baselines attain better total energy performance when compared to NSGA-II and MFA as a result of adaptive offloading; however, exploration dynamics and cumulative training costs upsurge overall system energy under heavy load. For real deployments, this implies extended node lifetime, enhanced thermal stability, and higher QoS without hardware scaling, making LITO appropriate for dense urban fog zones and industrial IIoT edges.

In Figure 11, although baselines show high utilization, they indicate localized saturation instead of balanced efficiency. LITO maintains lower energy and network overhead while preserving balanced resource utilization across fog layers. Moreover, LITO attains stable, non-congestive utilization when compared to distributed RL methods by distributing tasks proportionally across edge and fog layers. This approach avoids premature resource exhaustion, maintaining SLA compliance.

### 5.2. Impact of Task Complexity

Figure 12 illustrates the comparative analysis of task latency across changing task complexities under Lightweight Edge Load with 100 tasks per second. It reveals the superior performance of the proposed LITO framework when compared to the baselines like NSGA-II and MFA. This is mainly due to LITO’s mechanism design that includes feasibility filtering minimizing wasted execution and queueing delays; distributed ED/SN/PN role-based selection avoids oscillations and minimizes offloading churn, and adaptive “huddling” behavior spreads overload across nearby nodes, identical to lemur thermoregulation that stabilizes service latency under spikes. Under high-complexity stress conditions, baselines collapse because of centralized decision bottlenecks and lack of overload diffusion, while LITO sustains QoS via balanced resource allocation along with intelligent offloading. Practically, this stability makes LITO appropriate for real-time, delay-sensitive edge applications.

Under increasing task complexities, LITO maintains service reliability and energy feasibility efficiently when compared to NSGA-II and MFA, proving stronger workload resilience in high-stress regions. Figure 13 supports this by highlighting that LITO consistently attains lower offloading ratios when complexity increases. This is because LITO utilizes role-based coordination between EDs and SNs to minimize oscillation and queue churn, feasibility screening at the PN to avoid non-beneficial offloading, and localized huddling to redistribute bursts of load. It ensures that remote execution is only triggered when net performance gains are anticipated. Therefore, LITO identifies scenarios in which local execution is energy-efficient, eliminating redundant remote offloading. In stress regions where baselines depend on aggressive offloading, they experience network contention and increased queuing delays, while LITO’s selective offloading of only high-impact tasks produces meaningful reductions in energy and latency. Practically, this allows stable QoS under rapidly increasing workloads without over-provisioning.

### 5.3. Impact of Workload Characteristics and Topology

**Figure 14:**
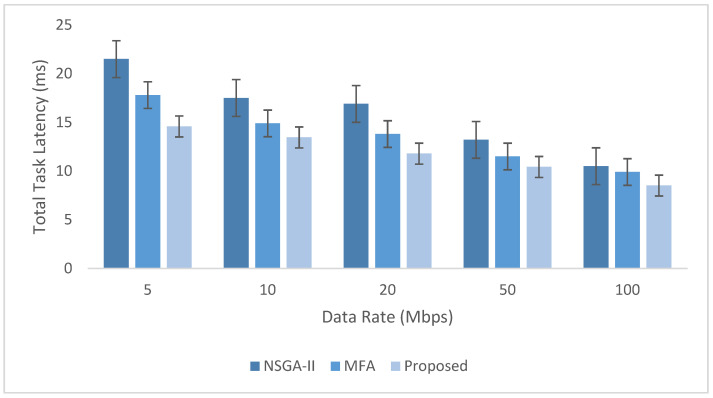
Comparison of task latency with respect to increasing data rates.

Table 4 evaluates the proposed LITO framework under increasing workload intensity across four topologies, reporting makespan, throughput, task completion time, computational cost, TSR, offloading ratio, and SLA violation rate. Table 4 reports LITO-only results to isolate topology and workload effects; baseline comparisons are provided in Figure 7, Figure 8, Figure 9, Figure 10, Figure 11, Figure 12, Figure 13 and Figure 14. The node configuration of the four topologies is given in Table 3.

Overall, the results indicate that LITO can handle heterogeneous fog resources while maintaining reliable and efficient operation as workload intensity changes. The observed stability under load is consistent with LITO’s mechanism-level design (e.g., feasibility-aware filtering and role-based task assignment) rather than ad hoc parameter tuning. In particular, throughput increases with workload up to moderate levels, reflecting effective scheduling and distribution of tasks across EDs and fog nodes. At extremely high workloads, throughput gains diminish in some configurations due to saturation and resource contention—an expected behavior as CPU capacity, bandwidth, and queue limits become binding constraints.

Importantly, LITO mitigates early congestion by filtering infeasible tasks at the ED level, preventing unnecessary forwarding that would otherwise amplify queue buildup and reprocessing. As a result, throughput remains stable until the system approaches hard saturation limits imposed by the underlying topology and resource capacities.

Makespan analysis shows that task completion times scale with workload size, underscoring the growing influence of processing and communication delays. The proposed LITO framework alleviates extended makespan through its hybrid scheduling strategy in which the DRS-based scheduling ensures fair resource allocation for normal tasks, and the EDF queue prioritizes latency-sensitive tasks. This hierarchical task scheduling aids the system in preserving responsiveness, particularly under high workloads, without disproportionately penalizing longer-running tasks. In addition, it reduces scheduling oscillation, prevents starvation among long-running tasks, and lowers queue time for urgent tasks. Similarly, computational cost remains controlled across different configurations, representing that the LITO algorithm effectively employs fog and edge resources to minimize overhead. This efficiency arises from the lemur-inspired huddling strategy, in which EDs implicitly collaborate by sharing overload via selective offloading instead of offloading indiscriminately. By distributing bursty workloads across multiple nodes, huddling stabilizes SLA performance during spikes, enhancing feasibility without overconsuming any single resource pool, which contrasts with traditional metaheuristic methods that may overload resources because of the lack of dynamic feasibility awareness.

Task completion time and task success ratios remain favorable when workload increases, while reflecting that tasks are completed successfully and within reasonable delay bounds. Lower task completion time is attained since LITO filters infeasible tasks at EDs and dispatches only schedulable workloads upward, eliminating redundant queueing and reprocessing at fog nodes. Task success ratios remain high under load by reflecting limited task abandonment and reliable execution. This integration indicates that tasks are both feasible and finishable instead of merely successful. This robustness leads to decentralized coordination among SNs, PNs, and EDs: SNs preserve neighborhood-level resource visibility, PNs perform global orchestration when necessary, and EDs decide feasibility locally. Such role separation suppresses oscillation between global and local decision scopes, minimizing both re-execution overhead and deadline violations.

The offloading ratio and SLA violation further emphasize the adaptability of the LITO framework. LITO minimizes offloading ratio under heavy workloads, eliminating deadline pressure and congestion at fog nodes. Configurations with very high workload sizes show modest SLA violation increases, which is expected in real deployments, yet overall rates remain low, demonstrating effective orchestration. Crucially, in stress cases where baseline methods would degrade severely due to unmanaged contention and queue saturation, LITO mitigates cascading failures through feasibility checks, role-based selection avoids churn between ED and fog layers, huddling spreads overload. This combination preserves SLA degradation by making degradation gradual rather than catastrophic, proving graceful failure behavior instead of abrupt collapse.

Task latency is measured under Lightweight Edge Load with a workload size of 100. Figure 14 illustrates that total task latency consistently decreases when data rate increases, indicating the reduced transmission time across the network. This superior performance arises from mechanism-level advantages of LITO. At low data rates, these mechanisms are vital since baselines confront sharp latency spikes because of uncoordinated task assignment and re-execution, whereas LITO preserves stable, low latency by adapting offloading decisions and task execution dynamically. Even as transmission delays reduce at higher data rates, LITO continues to surpass others, while ensuring steady end-to-end latency reduction under both stress and nominal conditions.

Attaining lower latency with LITO necessitates slightly higher energy consumption because of role-based coordination and dynamic task migrations. Likewise, improved SLA is associated with a lower offloading ratio, since feasibility checks avoid overloading edge nodes. In other words, moderate energy overhead and reduced offloading flexibility are sacrificed to gain more reliable SLA and stable latency, emphasizing LITO’s balanced approach to performance optimization.

### 5.4. Ablation Study on Execution Scenarios

**Figure 15:**
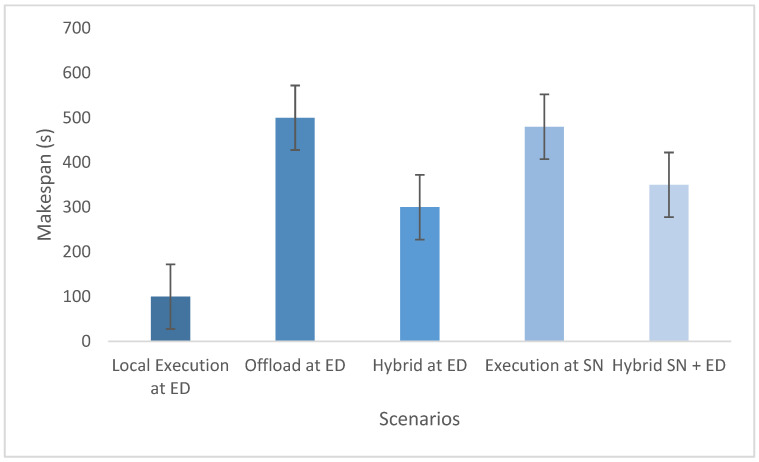
Comparison of makespan across different execution scenarios.

**Figure 16:**
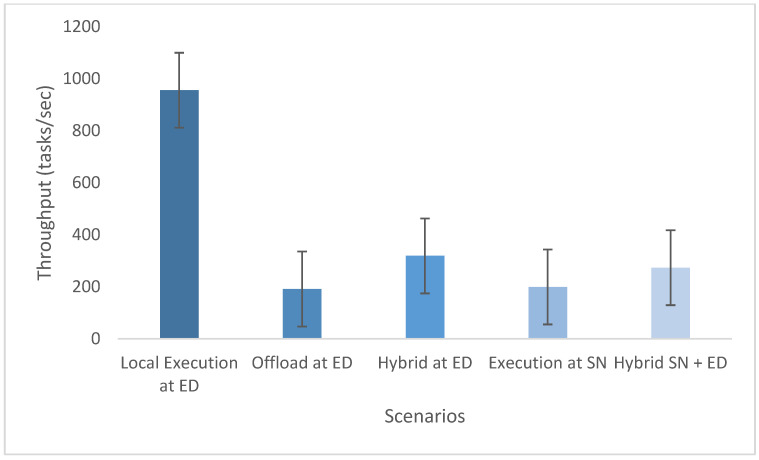
Comparison of throughput across different execution scenarios.

Figure 15 and Figure 16 illustrate the effect of diverse execution strategies on makespan and throughput within a fog-computing environment. Executing tasks only at ED leads to the lowest makespan and highest throughput because local processing avoids offloading delays and offloading congestion. However, this approach risks resource saturation under high-load conditions, resulting in potential queuing and SLA violations. On the other hand, offloading all tasks from the ED results to fog nodes in a significantly higher makespan and a sharp reduction in throughput as a result of communication overhead, task oscillation across SNs and PNs, and congestion at fog nodes, demonstrating that purely centralized execution is not suitable for edge-heavy traffic. The hybrid-ED strategy, in which tasks are partially processed locally and partially offloaded, offers a balanced behavior. Makespan modestly increases when compared with pure local execution, but throughput stabilizes since tasks are dynamically distributed across nodes, mitigating overload conditions while effectively utilizing available fog resources. By utilizing role-based selection and huddling mechanism, the proposed hybrid SN + ED model further improves performance. Huddling spreads tasks amongst lightly loaded EDs and SNs, minimizing oscillation and mitigating sudden increases in queue lengths. The basking (energy-aware) rule guarantees that tasks are executed with priority in which energy consumption is minimal without affecting task latency.

**Figure 17:**
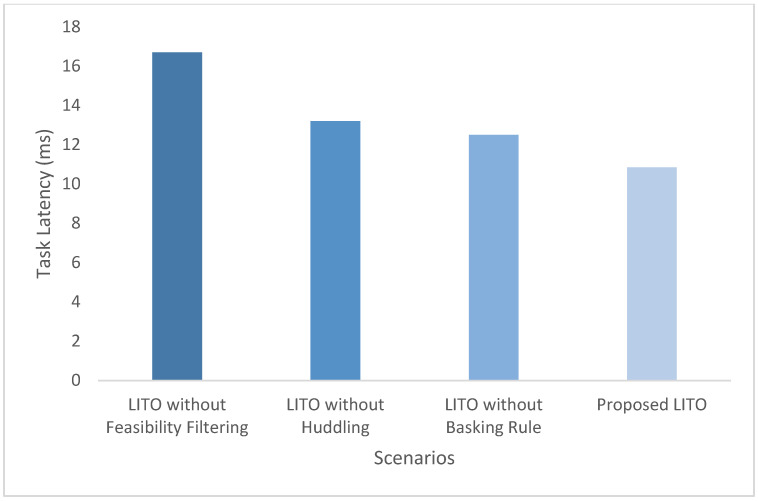
Comparison of task latency for the LITO ablation study.

Figure 17 confirms that these mechanisms’ contributions (the full LITO model) achieves the lowest latency. Removing huddling rises latency, since load is unevenly distributed, leading to queuing delays. Disabling basking further increases latency due to less energy-efficient execution decisions. In contrast, removing feasibility filtering yields the highest latency, since tasks are allocated to overloaded nodes, inducing re-execution and congestion. These results prove that the combination of lemur-inspired mechanisms allows LITO to preserve performance under high-load stress, in which baseline strategies collapse, while ensuring minimized queuing, balanced workload distribution, and stable SLA adherence.

### 5.5. Robustness Evaluation Under Dynamic Disturbances

To assess robustness under realistic fog disturbances, controlled disturbance events are inserted during simulation runtime. For the dynamic node failure scenario, one or two SNs are randomly deactivated at a uniformly sampled time between 30% and 60% of the total simulation period. During failure, the PN instantly eliminates the failed SN from the action set, and all newly arriving tasks are reallocated through the fallback routing mechanism and the feasibility filter. Tasks queued at the failed SN are redistributed to the following best feasible SN on the basis of CSPL-CB policy. For network perturbation experiments, link bandwidth between IoT–ED and PN–SN layers is changed dynamically within ±30% of nominal values through a stochastic perturbation model, and random latency spikes of 1–5 ms are also inserted to emulate transient congestion. The PN continuously updated the EWMA-based link statistics in X_t, enabling adaptive routing under unstable network conditions.

The robustness analysis in Table 5 shows that LITO demonstrates controlled degradation during dynamic disturbance conditions. When a single SN fails, system throughput confronts only a slight decline, which is accompanied by a minor upsurge in SLA violation rate and a small rise in makespan. This limited effect shows the efficiency of feasibility filtering, fallback routing, and hierarchical coordination at the PN. Under dual SN failures, performance degradation is proportional rather than severe, with moderate drops in throughput and manageable upsurges in SLA violations and completion time, signifying its improved operational stability and reduced processing capacity. In bandwidth perturbation scenarios, reduced bandwidth results in a modest performance impact as a result of increased transmission delays, whereas bandwidth improvement lowers makespan and improves throughput. Hence, LITO preserves stable TSRs and bounded SLA violations across all disturbance settings, proving its resilience and adaptive decision-making capability under dynamic fog environments.

## 6. Conclusions

This study addressed persistent challenges in fog computing, including energy inefficiency, latency, and uneven resource utilization. By modelling fog-layer coordination through cooperative lemur social behaviors, LITO adopts a decentralized architecture in which EDs, SNs, and PNs collaboratively manage workload distribution. The framework supports local decision-making via VFDT-based incremental learning to adapt to workload changes, while CSPL-CB policy refinement enables PNs to adjust task distribution across the fog hierarchy. At the SN layer, hybrid EDF-DRS scheduling prioritizes latency-sensitive tasks while maintaining efficient allocation for normal workloads.

Experimental results across multiple configurations, workload sizes, and task-complexity settings show that LITO reduces communication and computation energy consumption, improves throughput and resource utilization, and strengthens SLA adherence compared with the evaluated baselines. Under extreme workloads, performance becomes bounded by resource contention and queueing limits; however, LITO maintains stable behavior and balanced utilization relative to competing methods. Overall, LITO provides a practical biologically inspired approach to adaptive task offloading in fog environments.

Future work will extend LITO by integrating predictive workload models that forecast arrival intensity and task complexity to enable proactive feasibility filtering and earlier congestion avoidance. It will also incorporate fault-tolerant offloading under node and link failures through redundancy-aware placement, re-routing, and graceful degradation policies. Finally, we will quantify end-to-end overhead and scalability, including decision latency and messaging cost, and validate the framework under more realistic dynamic conditions such as time-varying bandwidth and mobility.

## Figures and Tables

**Table 1:** Summary of well-known metaheuristics-based task offloading approaches.

Ref.	Optimization Objectives	Decisions Metrics	Algorithmic Strategy	Centralized/Decentralized	Dynamic Adaptation Capability	Application Domain	Limitations
[21]	Joint energy-efficient task offloading and UAV trajectory optimization	Energy consumption, amount of offloaded data, maneuverability, and obstacle avoidance	Hybrid alternating metaheuristics	Centralized	Static	UAV-assisted MEC	Limited scalability: task and device heterogeneity are not addressed
[24]	Multi-objective workflow scheduling optimization	Average task delay, energy cost, workload distribution	Quantum-Inspired PSO	Centralized	Static	Real-time MEC with variable workloads	Static PSO parameterization
[25]	Task offloading optimization in fog/IoD	Transmission delay, fog computing delay, storage capacity, processing capacity	PSO	Centralized	Partial (iterative but not environment-adaptive)	IoD	PSO is susceptible to premature convergence and local optima
[26]	Multi-objective task offloading with Digital Twin integration	Task execution time, bandwidth, server capacity, and device energy consumption	PRLS, WCM	Centralized	Partially dynamic (state-aware iterative optimization)	Industrial IoT	Fixed parameters are considered
[27]	Latency-aware hybrid offloading optimization	Latency, load balancing, and task offloading time	FSA, HBA	Centralized	Static	Latency-sensitive IoT	More complex due to two-stage optimization
[28]	Joint resource utilization and delay minimization	Delay, resource utilization, task failure handling	DJA	Centralized	Partial (limited dynamic capability)	IoT–fog computing	NP-hard nature makes convergence slow
[30]	Multi-objective system-wide offloading optimization	Response time, energy consumption, cost, and availability criteria	INSCSA	Centralized	Static	Fog computing	Lack of consideration of dynamic workloads
[31]	Joint offloading and scheduling optimization	Energy consumption, transmission delay, task priority	MoAOA	Centralized	Static	Real-time IoT with cloud–fog	High computational complexity
[29]	Dynamic offloading under OFDM	Delay, energy consumption	Parallel Multi-threaded PSO	Decentralized	Dynamic	IoT-fog	Lack of dynamic condition handling
[45]	Pareto-based makespan–cost optimization	Makespan, cost	LD-NPGA	Centralized	Static	IoT-fog	Generate diverse Pareto sets while increasing decision complexity
[32]	Hierarchical latency and energy optimization	Delay, resource, and energy utilization	GA-SA-GWO	Centralized	Dynamic	Edge-fog	Computational complex; slow offloading decisions
[33]	Hybrid fog-based latency optimization	Transmission delay, delay, storage capacity	Genetic Algorithm	Centralized	Static	IoD-fog	Task prioritization and heterogeneity are ignored
[34]	Latency reduction in IoT applications	Latency, offloading time	SACO	Centralized	Static	IoT-fog computing	Performance relies on parameter tuning
[35]	Multi-objective scheduling in workflows	Makespan, cost, resource utilization	HAS, Genetic algorithm	Partially Decentralized	Static	Scientific workflows; fog-Cloud	Lack of adaptivity to dynamic workloads
[36]	Multi-objective critical task offloading	Energy consumption, delay, resource utilization, server availability	MFA	Centralized	Partial dynamic	Edge-Fog-Cloud	Limited consideration of heterogeneity
[37]	Multi-strategy offloading optimization	Execution time, operating cost, and convergence	lMA	Centralized	Partial dynamic	IoT; cloud–fog	Focus only on a single objective
[39]	Delay and energy-aware optimization	Power consumption, delay, and offloading probability	NSGA-II; Bees Algorithm	Centralized	Static	IoT-fog	Computationally expensive clustering dependency
[42]	Delay and energy-aware distributed load balancing optimization	System cost, latency, energy consumption, resource utilization	Distributed Twin-Delayed DDPG	Decentralized	Dynamic	MEC-enabled vehicular/fog computing	Training instability under extreme non-stationarity
[43]	Multi-objective critical task offloading	Execution time, convergence speed, scalability, decision time overhead	Asynchronous PPO with V-trace and PPO clipping	Decentralized	Dynamic	Fog computing	Higher decision time overhead
[44]	Multi-strategy delay-minimization and mobility-aware offloading optimization	Task delay, completion rate, energy consumption	Actor–Critic DRL (DDPG)	Decentralized	Partially dynamic	Heterogeneous MEC	Less stable convergence

**Table 2:** Mapping lemur behaviors to the LITO framework.

Algorithm	Lemur Behavior (PLBA)	LITO Mechanism
System Objective	Adaptive group survival and efficiency	Adaptive and efficient task offloading in fog systems
Hierarchy/Roles	Females (leaders), Males (supporters), Offspring (learners)	PNs (global coordinators), SNs (overflow handlers), EDs (local learners and executors)
Inputs	Environmental stimuli (threats, temperature, food), internal energy/health state	Resource availability, task urgency, energy levels
Monitoring Mechanism	Continuous observation of surroundings and internal status	Real-time sensing of network and device conditions
Decision Flow	Female-led decisions, male support, offspring follow and learn	PN-led routing, SN task support, ED local decision-making and learning
Energy Restoration (Sun Basking)	Seeking sunlight to replenish energy	Offloading tasks to resource-rich nodes to optimize energy usage
System Stability (Huddling)	Clustering for warmth and protection under stress	Cooperative task redistribution during heavy load or node failures
Learning Mechanism	Offspring observe adult behavior to develop survival strategies	EDs incrementally learn using VFDT and performance feedback
Execution Outcome	Coordinated foraging, relocation, and defense	Distributed task allocation ensuring low latency, energy efficiency, and SLA adherence

**Table 4:** LITO results showing the impact of varying IoT, ED, SN, and PN configurations on diverse metrics.

Configurations/Topology	Workload Size (tasks/s)	Throughput (tasks/s)	Makespan (s)	Comp. Cost (G$)	Task Completion Time (ms)	Task Success Ratio	Offloading Ratio	SLA Violation Rate
Lightweight Edge Load	60	52	8.389	139,680.0	19.4	0.97	2.4%	0.03
	120	108	9.758	141,380.0	21.1	0.96	1.2%	0.04
	180	158	9.091	144,520.0	23.5	0.95	0.8%	0.05
Moderate Edge Load	100	92	18.056	149,280.0	22.2	0.94	1.4%	0.06
	200	182	19.228	153,380.0	25.8	0.93	0.7%	0.07
	300	270	20.732	158,760.0	29.6	0.91	0.5%	0.09
Dense Edge Load	180	163	48.072	166,920.0	33.8	0.88	0.8%	0.11
	360	318	53.35	173,280.0	37.2	0.86	0.4%	0.14
	540	468	58.629	181,520.0	41.9	0.83	0.3%	0.17
High-Density Edge Load	300	245	164.929	192,440.0	49.3	0.80	0.4%	0.19
	600	465	171.872	205,760.0	56.7	0.76	0.2%	0.23
	900	675	182.767	221,840.0	63.9	0.72	0.1%	0.26

**Table 5:** Robustness evaluation of LITO under bandwidth perturbations and dynamic SN failures.

Scenarios	Throughput	SLA Violation Rate (%)	TSR	Makespan
Normal	270	0.09	0.91	20.732
1 SN Failure	263.5	0.101	0.899	21.210
2 SN Failure	257.8	0.112	0.888	21.964
Bandwidth −30%	259.2	0.105	0.895	21.650
Bandwidth +30%	278.4	0.082	0.918	20.118

## Data Availability

No new data were created or analyzed in this study. Data sharing is not applicable to this article.
